# Prognostic factors and effect modifiers in patients with relapsed or refractory follicular lymphoma who failed at least two lines of therapy: a systematic literature and expert clinical review

**DOI:** 10.1007/s00277-025-06575-9

**Published:** 2025-09-02

**Authors:** Pau Abrisqueta, Ana Jiménez-Ubieto, Ángel Serna, Irene Zamanillo, Yuting Kuang, Jennifer Uyei, Mohsin Shah, Laura Walsh, Eileen Thorley, Krystal Cantos, Emaan Rashidi, Qiufei Ma, Jessica J. Jalbert, Alexi N. Archambault, Yingxin Xu, Shivani Aggarwal, Srikanth Ambati, Hesham Mohamed, Christian Hampp, Bastian von Tresckow

**Affiliations:** 1 Department of Hematology, Hospital Vall d’Hebron, Vall d’Hebron Institute of Oncology (VHIO), Barcelona, Spain; 2 Hematology Department, University Hospital 12 de Octubre, Madrid, Spain; 3 IQVIA, Inc., Durham, NC USA; 4 Regeneron Pharmaceuticals, Inc., Tarrytown, NY USA; 5 Department of Hematology and Stem Cell Transplantation, West German Cancer Center, and German Cancer Consortium (DKTK partner site Essen), University Hospital Essen, University of Duisburg-Essen, Essen, Germany

**Keywords:** Relapsed/refractory follicular lymphoma, Prognostic factors, Systematic literature review, Expert clinical review, Real-world data-derived external control arms, Externally controlled trials

## Abstract

To enhance the quality of real-world external comparative studies, it is essential to systematically identify, prespecify, and account for prognostic variables and effect measure modifiers (EMMs), especially between emulated target trial and real-world control arms. These factors can then be utilized to evaluate cohort comparability and perform covariate adjustments, such as in propensity score models. A systematic literature review (SLR)-based identification of prognostic factors, coupled with expert clinical review, offers a comprehensive approach to evaluating and ranking the level of evidence, while also assisting in selection of prognostic factors to assess imbalances between cohorts in single-arm trials and real-world data studies. We performed an SLR followed by a clinical review and ranking by subject-matter experts to identify prognostic factors and EMMs in patients with relapsed or refractory (r/r) follicular lymphoma (FL) who failed at least two lines of therapy (LoTs). Across 13 included studies, the SLR identified 28 prognostic factors that were significantly associated with clinical outcomes, including overall survival, progression-free survival, and objective response rate. Notably, our review did not identify any statistically significant EMMs. Based on expert ranking of the SLR-derived list, the 5 most important prognostic variables in descending order are: progression of disease within 24 months of first LoT (POD24), chemo-immunorefractory/chemoresistant, refractory to last LoT, number of prior LoTs, and serum lactate dehydrogenase. This comprehensive SLR and expert review highlight critical prognostic factors in r/r FL. The identified prognostic variables can inform future research, emphasizing the need for continued investigation into factors affecting outcomes in this challenging and heterogeneous patient population.

**Supplementary Information:**

The online version contains supplementary material available at 10.1007/s00277-025-06575-9.

## Introduction

Follicular lymphoma (FL) is the most common type of indolent non-Hodgkin lymphoma (NHL), which accounts for 17–35% of all NHL cases in the United States and Europe [1, 2]. Approximately 20% of patients with FL are expected to relapse within 2 years of initial treatment [3, 4]. There is no established standard of care for treating early relapsed or refractory (r/r) FL, and the response to these treatments is often variable and suboptimal [5, 6]. Considering the cumulative toxicity, limited treatment options, and unfavorable outcomes for patients requiring multiple treatment lines, there is a significant unmet need for effective treatments for patients with r/r FL, particularly those requiring third-line or later (3 L+) therapies.

The most significant clinical progress in the therapeutic arena has been in the field of immunotherapy, with novel treatments including chimeric antigen receptor (CAR) T-cell therapy and T-cell–engaging bispecific agents. These therapies have altered the treatment landscape for r/r FL with proven efficacy and an acceptable safety profile. However, a substantial number of patients are likely to experience repeated relapses, with an increasing resistance to treatment over time [7]. Additional research is required to determine the most effective way to employ CAR T-cell therapy and T-cell–engaging bispecific agents in individuals with r/r FL, with the possibility of achieving a cure.

Increasingly, real-world data (RWD)-derived external control arms have been used to contextualize single-arm clinical trials as supportive evidence of treatment effectiveness in regulatory and payer decision-making. In such studies, prespecification of prognostic factors for adjustment is required [8, 9]. Combining the identification of prognostic factors and effect measure modifiers (EMMs) through a systematic literature review (SLR) with clinical expert input is a comprehensive approach to select variables a priori for confounder adjustment in comparative analyses. This approach is aligned with guidance provided by the Institute of Quality and Efficiency in Health Care (IQWiG), which requires relevant confounders to be systematically identified on the basis of scientific literature, with the involvement of subject experts, and prespecified in the study protocol [10]. This approach can be used in comparative evaluations of treatment effectiveness and safety. Finally, this approach can be employed for adjusting estimates using propensity score models and evaluating balance in key patient characteristics in comparative studies, including when comparing single-arm trial populations with RWD-derived external control arms, to ensure exchangeability.

The main objective of this SLR combined with expert clinical review was to identify and rank prognostic factors and EMMs systematically and comprehensively in adult patients with r/r FL grade 1–3a who failed at least two prior lines of therapy (LoTs).

## Methods

The research was carried out in two stages. In the first stage, we conducted an SLR to identify potential prognostic factors, and in the second stage, subject-matter experts conducted a clinical evaluation to contextualize these findings. The SLR followed guidelines set forth by the Cochrane Handbook for Systematic Reviews of Interventions [11] and Preferred Reporting Items for Systematic Reviews and Meta-Analyses (PRISMA) [12]. Guidelines from the European Medicines Agency (EMA) [13], US Food and Drug Administration (FDA) [14], IQWiG [15], and UK National Institute for Health and Care Excellence (NICE) [16, 17] were also reviewed for SLR methodology, as applicable.

A detailed protocol was developed prior to conducting the review, and the review was registered prospectively in PROSPERO (registration ID CRD42022307561).

### Search strategy

Comprehensive literature searches were conducted using Medical Literature Analysis and Retrieval System Online (MEDLINE), Excerpta Medica Database (Embase), and Cochrane Central Register of Controlled Trials (CENTRAL) between January 1, 2016, and December 13, 2021 (complete search strategies are presented in Appendix A). Searches were supplemented by conference abstract reviews for the American Society of Clinical Oncology (ASCO), European Society for Medical Oncology (ESMO), American Society of Hematology (ASH), and European Hematology Association (EHA) conferences in 2021. Forward citation searches were undertaken using Google Scholar based on 10 included references. The bibliographies of four recently published reviews on the related topic area, as well as ESMO and National Comprehensive Cancer Network (NCCN) guidelines, were also reviewed to identify additional relevant studies [18–21].

### Eligibility criteria

The scope of the research and patient, intervention, comparison, outcome, time and setting (PICOTS) criteria for including and excluding studies are outlined in Table 1. Studies were eligible for inclusion if they included adults (18 years or older) with r/r FL grade 1–3a who failed at least two LoTs and initiated a subsequent treatment (3 L+). There were no restrictions for interventions or comparators. Clinical trials or observational studies reporting on potential prognostic factors or EMMs that were associated with objective response rate (ORR), overall survival (OS), progression-free survival (PFS), time to next treatment, complete response (CR) rate, duration of response, disease control rate, or histologic transformation were included.

**Table 1:** PICOTS criteria

Criteria	Description
Populations	Adult patients with r/r FL grade 1–3a who failed at least two LoTs (3 L+) Other applicable eligibility criteria: • Lymphoma type: Include only studies with 100% patients with FL or if results were stratified for FL; exclude studies with mixed lymphoma types where results were not stratified • LoT: Include studies where at least 50% of patients received 3L + therapy (i.e., median or mean of at least two prior LoTs); exclude studies that did not report the number of prior LoTs
Interventions	• Any or none
Comparators	• Not applicable
Outcomes^a^	• Potential prognostic factors^b^ or effect measure modifiers^c^ that were associated with ORR, OS, PFS, TTNT, CR, DOR, DCR, or HT
Time	• Publication date limit: January 1, 2016, to December 13, 2021
Study design	• Include: RCT, nonrandomized trial, observational study • Exclude: Case reports, evidence synthesis studies or reviews (flag for bibliography), health economic modeling/economic/resource use studies
Other	• Exclude: Nonhuman, pediatric/pregnancy; publication type as editorials, letters, notes, commentaries • Geography: Global • Language: English (journal article or conference abstract)

*3L +* third line or later; *CR* complete response; *DCR* disease control rate; *DOR* duration of response; *FL* follicular lymphoma; *HT* histologic transformation; *LOT* line of therapy; *ORR* overall response rate; *OS* overall survival; *PFS* progression-free survival; *PICOTS* patient, intervention, comparison, outcome, time and setting; *RCT* randomized controlled trial; *r/r* relapsed or refractory; *TTNT* time to next treatment

^a^Notes for outcomes: (1) The search and screening were kept broad in order to capture studies reporting on prognostic factors, predictive factors, correlation, association, confounders, effect measure modifiers, subgroups, and other related concepts; (2) information was extracted for the statistically significant variables only. If multiple models are reported within a study, results were extracted from the most adjusted model. Studies were excluded if statistical significance was not concluded for any model variables

^b^Defined as variables, including confounders, that are associated with subsequent health outcomes among people with a particular health condition

^c^Defined as factors that modify the effect of the putative causal factor(s) under study; effect measure modification occurs when the magnitude of the effect differs depending on the level of a third variable

### Study selection, data collection, and risk of bias assessment

The search process involved identifying unique records, which were then screened for eligibility by two independent reviewers. Any records with uncertain inclusion/exclusion criteria and any discrepancies between the reviewers were adjudicated by a third reviewer. The entire process was summarized using a PRISMA flow diagram. Eligible studies were selected, and their data were compiled in an Excel spreadsheet for synthesis. For each study, investigators identified key methodologic characteristics, patient characteristics, and results, and these data were extracted and tabulated. To ensure accuracy, numeric values were extracted independently by two reviewers, and checked against the source document by a third reviewer. Only variables and clinical outcomes with statistically significant associations (*p* < 0.05) were extracted. Risk of bias assessment of individual studies was performed using the Quality in Prognostic Studies (QUIPS) tool [22] (Appendix B). Potential threats to validity were assessed within six domains: (1) study participation; (2) study attrition; (3) prognostic factor measurement; (4) outcome measurement; (5) study confounding; and (6) statistical analysis and reporting.

### Data synthesis

All eligible studies were included to describe the prognostic factors and/or effect modifiers reported for individual clinical outcomes. Results were synthesized narratively by the type of prognostic factors, with findings tabulated.

### Clinical review and consensus process

Following the conduct of the SLR, the identified potential prognostic variables were evaluated by the study team to only include baseline variables, and therefore remove outcome (e.g., interval between frontline treatment and the second relapse [PFS2]) and treatment-specific (e.g., graft-versus-host disease [GVHD]) variables, determine their availability in a single-arm trial (ELM-2 [23]) and in RWD, and develop a questionnaire (15 variables were removed, 3 variables were revised, and 7 variables were added [the variables are outlined in Appendix C]).

In the questionnaire (Appendix D), prognostic variables were grouped by type of variable: patient demographics and clinical characteristics; disease characteristics; prior treatment characteristics; imaging and laboratory measures. Each prognostic variable was reviewed by an international panel consisting of three clinical experts in the field of lymphoma who categorized the prognostic impact on treatment response and survival on a 5-point scale ranging from “very high importance” to “not important”. A holistic approach was taken for the ranking of variables (i.e., clinical experts were asked to “categorize them in terms of their prognostic impact on treatment response and survival”). The clinicians were asked to consider possible correlation among the variables, possible effect modifiers, specific variable definitions (e.g., early chemoimmunotherapy failure), and whether there were any other prognostically important variables not captured in the questionnaire. For each variable, the clinical experts categorized the availability within RWD on a 3-point scale ranging from “readily available” to “limited availability”.

Questionnaires completed by the three clinical experts were evaluated, and the 10 most important variables were identified by summing the clinicians’ categorization of prognostic impact and considering variable availability in the event of a tie. Individual interviews were conducted with each clinical expert to clarify the variables and definitions, discuss discrepancies in categorization, and determine the prognostic variables’ rankings from 1 to 10. After the interviews, the ranking of each variable was summed across the three clinical experts to determine the final ranking. In the event of a tie the variables were assigned the same rank.

## Results

### Studies identified

**Fig. 1:**
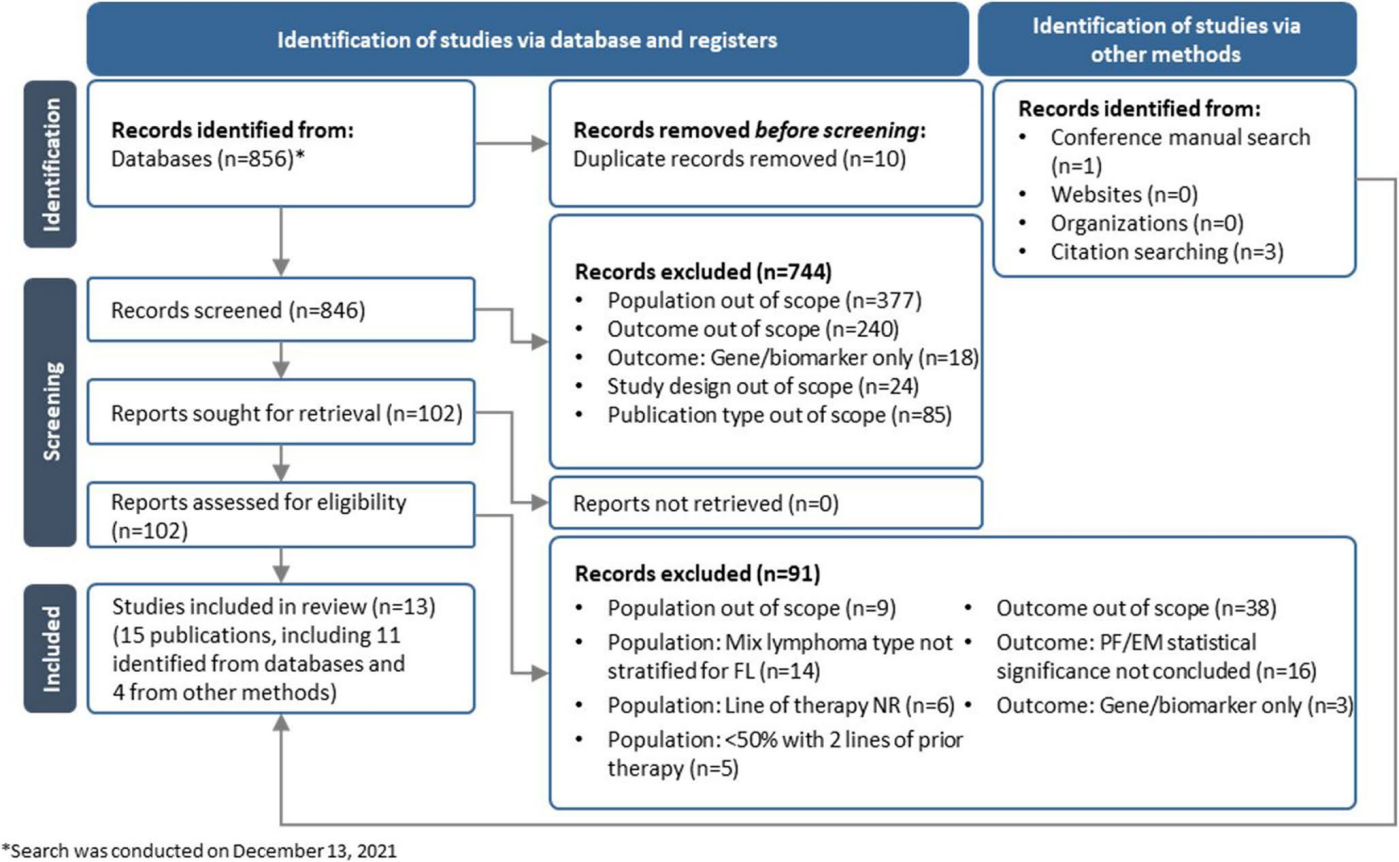
PRISMA flow diagram. *FL*, follicular lymphoma; *NR*, not reported; *PF/EM*, prognostic factors/effect modifiers; *PRISMA*, Preferred Reporting Items for Systematic Reviews and Meta-Analyses

The database searches identified a total of 856 records. Following deduplication, 846 records underwent title and abstract screening, of which 102 records were retained for full-text review. After full-text review, 11 records [24–34] meeting the eligibility criteria were included. Four additional records [35–38] were identified by other methods. Overall, 15 publications (nine journal articles and six conference abstracts) reporting data on 13 studies were included in the review (Fig. 1).

### Study and patient characteristics

Characteristics of the included studies are summarized in Supplementary Table 1 of Appendix E. Among the 13 included studies, 11 were observational studies based on data from clinical centers or registries including the European Society for Blood and Marrow Transplantation (EBMT) and Center for International Blood and Marrow Transplant Research (CIBMTR) [25–32, 36–38] and two were nonrandomized trials [24, 35]. In this review, clinical trials and observational studies were considered of equal grade in the evidence synthesis. Some studies included patients from overlapping data sources, but all were included to capture important subpopulations and to ensure thoroughness. The sample size of included studies varied from 40 patients [24] to 1567 patients [31], with median age ranging from 45 years [32] to 64 years [24] and median follow-up time spanning 17 months [35] to 140 months [25]. The interventions reported in the studies included stem cell transplantation in nine studies [25, 26, 29–32, 36–38], CAR T-cell therapy in one study [35], targeted therapy (ibrutinib) in one study [24], and chemotherapy in one study [28]. One study [27] did not report the intervention. In six studies [27–30, 32, 36], all patients with FL failed at least two prior LoTs. In the remaining seven studies [24–26, 31, 35, 37, 38], at least 50% of the study population received at least two prior LoTs (three studies) or had a median/mean of at least two prior LoTs (four studies). All studies included 100% patients with FL. Out of 13 studies, four studies were multicountry [24, 31, 32, 35] and two were conducted in the United States [30, 38], two in Japan [28, 37], two in Spain [27, 36], one in Germany [25], one in Poland [26], and one in France [29].

### Quality assessment of included studies

**Fig. 2:**
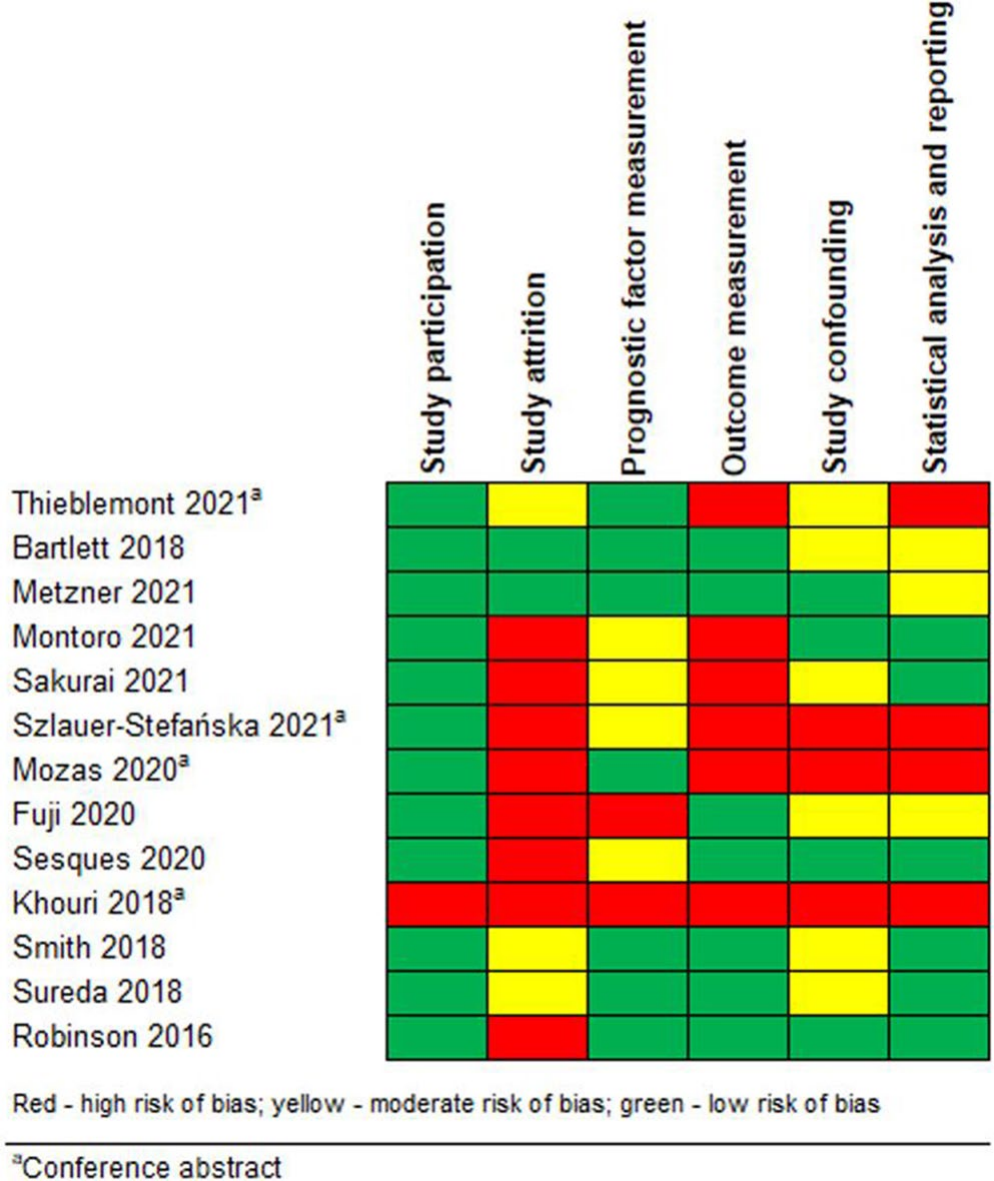
Risk of bias for prognostic factor studies

Results of the risk of bias assessment are displayed in Fig. 2. Risk of bias was assessed using the QUIPS tool [22]. In most of the studies, the risk of bias assessment for prognostic factor studies showed a lack of reporting, specifically in the “study attrition” and “study confounding” domains. Few studies posed a low risk of bias.

### Prognostic factors and EMMs

#### SLR

Across the 13 studies included in the SLR, 28 prognostic factors were identified that had statistically significant associations with the clinical outcomes of interest. Seven clinical outcomes with statistically significant associations (*p* < 0.05) were identified (most commonly OS, PFS, and relapse/progression). None of the studies identified statistically significant EMMs. The seven clinical outcomes with statistical associations were as follows: OS (in nine studies); PFS (in eight studies); relapse/progression (in four studies); non-relapse mortality (in four studies); transplant-related mortality (in one study); CR (in one study); and ORR (in one study).one

The prognostic variables were categorized into four groups: patient demographics and clinical characteristics; disease characteristics; prior treatment characteristics; and imaging and laboratory measures. The association directionality, associated clinical outcomes, and study counts for each prognostic factor are summarized in Tables 2, 3 and 4 (supporting results are presented in Supplementary Table 2 of Appendix E). Among identified variables, eight variables (older age, chemorefractory/chemoresistant disease, a greater number of prior LoTs, a lower Karnofsky performance status, a high-risk Follicular Lymphoma International Prognostic Index [FLIPI] composite score, not achieving complete response/partial response at transplant, use of myeloablative conditioning regimen, and a higher grade of GVHD) were associated with worse clinical outcomes in at least two studies. Other patient clinical, disease, and treatment characteristics, as well as laboratory measure variables, were identified from single studies. No variables demonstrated inconsistent directionality of association with clinical outcomes.

**Table 2:** Patient demographics and clinical characteristics – summary of study count, directionality, example characteristics, and affected outcomes for statistically significant prognostic factors

Prognostic factor	Study count	Directionality – characteristics associated with worse outcomes	Example characteristics (vs. reference) – category with favorable outcomes in bold	Clinical outcomes with study counts
Age	*N* = 3^a^	Older age	• Per year of age (as continuous variable) • ≥45 years (vs. **<45 years**) • >50 years (vs. **≤50 years**)	OS: 2^a^, PFS: 2, NRM: 3^a^, TRM: 1, relapse/ progression: 1
KPS	*N* = 2^a^	Lower KPS	• <80 (vs. **≥80**) • < 90 (vs. **≥90**)	OS: 1, PFS: 1, NRM: 2^a^, TRM: 1
ECOG PS	*N* = 1	Higher ECOG PS	• 2–4 (vs. **0–1**)	OS: 1, PFS: 1
HCT-CI^b^	*N* = 1	Higher HCT-CI	• High (vs. **low**)	PFS: 1, NRM: 1

*ECOG* Eastern Cooperative Oncology Group performance status; *HCT-CI* Hematopoietic Cell Transplantation Comorbidity Index; *KPS* Karnofsky performance status; *NRM* nonrelapse mortality; *OS* overall survival; *PFS* progression-free survival; *TRM* transplant-related mortality

^a^Two studies used the same data source and may have overlapping populations

^b^The HCT-CI is a comorbidity index that comprises 17 different categories of organ dysfunctions, including arrhythmia, cardiac comorbidity, inflammatory bowel disease, diabetes, cerebrovascular disease, psychiatric disturbance, mild hepatic comorbidity, obesity, infection, rheumatologic comorbidity, peptic ulcer, moderate/severe renal comorbidity, moderate pulmonary comorbidity, prior solid tumor, heart valve disease, severe pulmonary comorbidity, and moderate/severe hepatic comorbidity

**Table 3:** Disease and treatment characteristics – summary of study count, directionality, example characteristics, and affected outcomes for statistically significant prognostic factors

Prognostic factor	Study count	Directionality – characteristics associated with worse outcomes	Example characteristics (vs. reference) – category with favorable outcomes in bold	Clinical outcomes with study counts
Chemo-sensitivity	*N* = 3^a^	Chemorefractory or chemoresistant disease	• Chemoresistant (vs. chemosensitive) • Chemorefractory (vs. chemosensitive) • Rituximab-refractory disease (vs. **rituximab-sensitive disease**)	OS: 2^a^, PFS: 2^a^, NRM: 2^a^, TRM: 1, ORR: 1, relapse/progression: 2^a^
Prior LoTs	*N* = 2	Higher number of prior LoTs	• 3–4 (vs. **1–2**) • ≥5 (vs. 1–2 or vs. **3–4**) • >3 (vs. **≤3**)	OS: 1, PFS: 2, NRM: 2, TRM: 1
FLIPI score	*N* = 2	High-risk FLIPI score	• High risk (vs. **low risk**) • High risk (vs. **low/intermediate risk**)	OS: 1, PFS: 1
Disease status at transplant	*N* = 2	Not achieving CR/PR at transplant	• No complete remission (vs. **complete remission**) • Others (vs. **CR/PR**)	PFS: 1, relapse/ progression: 1
Conditioning regimen	*N* = 2	The use of myeloablative conditioning regimen	• Myeloablative (vs. **reduced intensity/nonmyeloablative**) • Myeloablative (vs. **reduced intensity**)	OS: 1, PFS: 2, TRM: 1
GVHD grade	*N* = 2	Higher grade of GVHD	• Acute II–IV (vs. **others**) • III–IV (vs. **I–II**)	OS: 1, NRM: 1
Histology	*N* = 1	Higher histology grade	• Grade 3 (vs. **grade 1**) • Missing (vs. **grade 1**)	OS: 1, PFS: 1, relapse/progression: 1
Ann Arbor stage	*N* = 1	Higher Ann Arbor stage	• III/IV (vs. **I/II**)	OS: 1
Disease stage at diagnosis	*N* = 1	Higher disease stage	• III/IV (vs. **I/II**)	Relapse/ progression: 1
Extranodal involvement at HCT	*N* = 1	Extranodal involvement	• Yes (vs. **no**) • Missing (vs. **no**)	OS: 1
Nodal sites involved	*N* = 1	Higher number of nodal sites	• ≥4 (vs. **<4**)	OS: 1
PFS2	*N* = 1	Shorter PFS2	• <2 years (vs. **>5 years or 2–5 years)**	OS: 1, CR after thirdline treatment: 1
POD24	*N* = 1	Presence of POD24	• Yes (vs. **no**)	PFS: 1
History of early treatment failure	*N* = 1	History of early treatment failure	• Yes (vs. **no**)	OS: 1
Duration of last remission prior to alloSCT	*N* = 1	Shorter duration of remission	• <1 year (vs. **>1 year**)	OS: 1
Time between ASCT and relapse	*N* = 1	Early relapse after ASCT	• <2 years (vs. >2 years)	OS: 1
Treatment line for ASCT	*N* = 1	Higher treatment line for ASCT	• Third/fourth (vs. **first**)	OS: 1
Histologic transformation at relapse after ASCT	*N* = 1	Histologic transformation at relapse after ASCT	• Yes (vs. **no**)	OS: 1

*alloSCT* allogeneic stem cell transplantation; *ASCT* autologous stem cell transplantation; *CR* complete response; *FLIPI* Follicular Lymphoma International Prognostic Index; *GVHD* graft-versus-host disease; *HCT *Hematopoietic Cell Transplantation; *LoTs* lines of therapy; *NRM* nonrelapse mortality; *ORR* overall response rate; *OS* overall survival; *PFS* progression-free survival; *PFS2* interval between frontline treatment and second relapse; *POD24* progression of disease within 24 months of first LoT; *PR* partial response; *TRM* transplant-related mortality

^a^Two studies used the same data source and may have overlapping populations

**Table 4:** Imaging and laboratory measures – summary of study count, directionality, example characteristics, and affected outcomes for statistically significant prognostic factors

Prognostic factor	Study count	Directionality –characteristics associated with worse outcomes	Example characteristics (vs. reference) – category with favorable outcomes in bold	Clinical outcomes with study counts
Hemoglobin	*N* = 1	Lower level of hemoglobin	• ≤12 g/dL (vs. >**12 g/dL**)	OS: 1, PFS: 1
LDH	*N* = 1	Elevated LDH	• High (vs. **normal**)	OS: 1, NRM: 1
Serum sIL2R level at third line	*N* = 1	Lower level of sIL2R	• ≤1080 IU/mL (vs. **>1080 IU/mL**)	PFS: 1
SUVmax in PET/CT	*N* = 1	Higher values of SUVmax high risk	• At cycle 1 day 8 PET/CT (as continuous variable) • At cycle 1 day 8 PET/CT ≥13.78 (vs. **<13.78**)	PFS: 1, ORR: 1
TMTV	*N* = 1	Higher TMTV	• High (>510 cm^3^, vs. **low**)	PFS: 1
Deauville score^a^	*N* = 1	Higher Deauville score	• ≥3 (vs. **<3**)	OS: 1

*LDH* lactate dehydrogenase; *NRM* nonrelapse mortality; *ORR* overall response rate; *OS* overall survival; *PET/CT* positron emission tomography/computed tomography; *PFS* progression-free survival; *sIL2R* soluble interleukin 2 receptor; *SUVmax* maximum standardized uptake value; *TMTV* total metabolic tumor volume

^a^The Deauville 5-point scale is based on a visual comparison between the uptake of lymphoma tissue and that of the liver and mediastinum in PET/CT

#### Clinical review

During the questionnaire and following individual interviews, no prognostic factors were considered to be missing by the clinical experts. All three clinical experts recommended the expansion of chemorefractory/chemoresistant to include chemo-immunotherapies. Regarding discrepant grading following the questionnaire, discussions were held with the clinical experts during the individual interviews.

The final ranked list of the 10 most important prognostic variables in descending order of importance determined following the individual interviews included: progression of disease within 24 months of first LoT (POD24); chemo-immunorefractory/chemoresistant; refractory to last LoT; number of prior LoTs; serum lactate dehydrogenase; Eastern Cooperative Oncology Group (ECOG) performance status; FLIPI; age at start of LoT; Ann Arbor disease stage; and refractory to rituximab (Table 5).

**Table 5:** Final ranked prognostic variables based on expert clinical review

Rank	Prognostic factor
1	POD24
2	Chemo-immunorefractory/chemoresistant
3	Refractory to last LoT
4	Number of prior LoTs
5	Serum LDH
5	ECOG performance status
7	FLIPI
8	Age at start of LoT
9	Ann Arbor disease stage
10	Refractory to rituximab

*ECOG* Eastern Cooperative Oncology Group; *FLIPI* Follicular Lymphoma International Prognostic Index; *LDH* lactate dehydrogenase; *LoT* line of therapy; *POD24* progression of disease within 24 months of first LoT

Variables were assessed prior to each LoT if not otherwise specified

## Discussion

RWD-derived external controls can be useful in contextualizing the effectiveness of single-arm trials when randomization is not feasible, impractical, or unethical. However, effectively contextualizing the findings of these trials using RWD requires identification of prognostic factors, potential EMMs, and prespecification of these variables for adjustment prior to conducting the analyses. An SLR combined with expert review can be a useful approach in these situations.

To our knowledge, this is the first study to use an SLR to identify prognostic factors in r/r FL, which were rigorously evaluated by subject-matter experts. Twenty-eight patient demographic, clinical, disease and treatment characteristics, and laboratory measures were determined as important prognostic factors of clinical outcomes for patients with r/r FL as reported in literature. However, no statistically significant EMMs were identified based on the SLR. Compared with prior reviews and published indices on the prognostic factors for FL [39, 40], this review confirmed that several patient demographic, clinical, disease, and treatment characteristics, as well as laboratory measures, are important prognostic factors for clinical outcomes in 3 L + r/r FL (grade 1–3a) patient populations and identified additional prognostic factors such as prior LoTs and ECOG performance status. Taking a holistic approach and to provide clinical context, an international panel of clinical experts reviewed and ranked the most significant SLR-derived prognostic variables. It was decided to include only one list of prognostic variables for all outcomes, consistent with previous publications [41, 42].

Regarding the operational aspects of conducting an SLR followed by expert clinical review, to ensure timeliness and efficiency, it is crucial to plan to run the review shortly after the SLR, given that scheduling challenges can introduce timeline risk. The results of the SLR should be reviewed in detail to ensure variable availability in the included RWD sources, prior to the solicitation of expert opinion. Lastly, a well-designed questionnaire accompanied by sufficient background study materials is required to ensure accurate and meaningful responses from the clinical experts. A strength of the approach taken with this expert clinical review – questionnaire followed by individual interviews – is that it has a mixed-methods research design, combining quantitative and qualitative approaches. This facilitates the consolidation of responses while allowing for an in-depth understanding of the clinical experts’ perspectives. Further, clinical experts from multiple countries were included to ensure consideration of multiple clinical experiences.

This study has certain limitations that should be noted when interpreting the results. First, for 20 of the prognostic factors, only one study reported a significant prognostic factor–clinical outcome association, and additional research is required to further validate the associations. Second, patient clinical and treatment characteristics, as well as treatment received prior to and during the study period, varied across the included studies. Although considered a strength of real-world evidence, the presence of heterogeneity has the potential to complicate interpretations of prognostic association estimates, particularly since not all patients were necessarily 3L+. Third, across studies, there was some overlap in data sources, which may cause some factors to be represented more than once and appear more important. Fourth, only variables and clinical outcomes with statistically significant associations (*p* < 0.05) were extracted. Given that statistical significance is highly influenced by sample and effect size, this study may not include an exhaustive list of every prognostic factor or EMM relevant to the patient population. Fifth, systematic reviews of published manuscripts can be susceptible to publication bias. To mitigate this bias, a comprehensive SLR strategy included searching informal sources such as conference and meeting abstracts. In addition, the risk of bias assessment showed a lack of reporting for the prognostic factor studies, specifically in the “study attrition” and “study confounding” domains. This may be due to insufficient reporting, especially in conference abstracts, and the fact that many prognostic factor analyses were exploratory in nature and not typically the primary objective of the included studies. Finally, while the study methods align with IQWiG guidance for systematic identification of relevant confounders and prognostic factors [10], the clinical review process remains inherently subjective, as the rankings were based on the opinions of three clinicians. Nevertheless, the insights provided by practicing clinicians added valuable perspectives on the clinical relevance of the identified prognostic factors, complementing the SLR, which was an objective process for identifying these factors. These rankings were not intended to inform clinical practice directly but could be considered alongside the SLR evidence during prognostic factor selection in future research.

## Conclusions

For real-world evidence generation, the selection of appropriate prognostic factors is crucial for valid outcome estimation. The findings of this study suggest that a multimethod approach combining an SLR-based identification of prognostic factors followed by expert clinical review provides comprehensive evaluation and ranking of the evidence to inform prognostic factor selection. These factors can be used for the evaluation of balance in key patient characteristics across RWD and single-arm trial cohorts, as well as for adjustment, for example, through their use in propensity score models for contextualizing outcomes of single-arm trials.

## Supplementary Information

Below is the link to the electronic supplementary material.

Supplementary Material 1

## Data Availability

For full transparency, we have made available in the manuscript and supplemental appendix: the search strategy, PRISMA flow diagram with reasons for exclusion, study level risk of bias, full list of references of included studies, table of study characteristics, and table of outcome data.
