# Supplementary material for: Prognostic factors and effect modifiers in patients with relapsed or refractory follicular lymphoma who failed at least two lines of therapy: a systematic literature and expert clinical review
Source: Ann Hematol. 2025 Sep 2;104(9):4357–67. doi: 10.1007/s00277-025-06575-9 (PMC12552330; doi:10.1007/s00277-025-06575-9)
Supplement: Supplementary file 1 — Supplementary Material 1 [file 277_2025_6575_MOESM1_ESM.docx]

**Prognostic factors and effect modifiers in patients with relapsed or refractory follicular lymphoma who failed at least two lines of therapy: a systematic literature and expert clinical review**

Pau Abrisqueta^1^, Ana Jiménez-Ubieto^2^, Ángel Serna^1^, Irene Zamanillo^2^, Yuting Kuang^3^, Jennifer Uyei^3^, Mohsin Shah^3^, Laura Walsh^3^, Eileen Thorley^3^, Krystal Cantos^3^, Emaan Rashidi^3^, Qiufei Ma^4^, Jessica J Jalbert^4^, Alexi N Archambault^4^, Yingxin Xu^4^, Shivani Aggarwal^4^, Srikanth Ambati^4^, Hesham Mohamed^4^, Christian Hampp^4^, Bastian von Tresckow^5^

**Affiliations**:

^1^ Department of Hematology, Hospital Vall d'Hebron, Vall d'Hebron Institute of Oncology (VHIO), Barcelona, Spain

^2^ Hematology Department, University Hospital 12 de Octubre, Madrid, Spain

^3^ IQVIA, Inc., Durham, NC, USA

^4^ Regeneron Pharmaceuticals, Inc., Tarrytown, NY, USA

^5^ Department of Hematology and Stem Cell Transplantation, West German Cancer Center and German Cancer Consortium (DKTK partner site Essen), University Hospital Essen, University of Duisburg-Essen, Essen, Germany

**Correspondence**:

Corresponding author name: Pau Abrisqueta

Affiliation: Department of Hematology, Hospital Vall d'Hebron, Vall d'Hebron Institute of Oncology (VHIO), Barcelona, Spain

Email: PAbrisqueta@vhio.net

**SUPPLEMENTARY APPENDIX**

**Appendix A - Search strategy for OVID-based searches**

Database(s):

EBM Reviews - Cochrane Central Register of Controlled Trials, November 2021

Embase, 1974 to 2021 December 10

Ovid MEDLINE(R) and In-Process, In-Data-Review & Other Non-Indexed Citations and Daily, 1946 to December 10, 2021

Date of search: December 13, 2021

| # | Searches | Results | Notes |
| --- | --- | --- | --- |
| 1 | Lymphoma, Follicular/ or follicular lymphoma/ or (Brill-Symmers or "nodular Lymphocytic Lymphoma*" or "nodular Histiocytic lymphoma*" or "Small Cleaved-Cell lymphoma*" or "follicle lympho*" or "follicular lympho*" or "nodular lymphoma*" or "nodular lymphomatos*").ab,ti. | 31824 | Population string – title/abstract/MeSH/Emtree field search  in adult patients with r/r FL, 3L+ therapies |
| 2 | treatment failure/ or (fail* or relapse* or refractory or resistan* or intolerant or progress).ab,ti. | 6537619 | population string - title/abstract/MeSH/Emtree field search |
| 3 | prognosis/ or prognostic assessment/ or confounding factors, epidemiologic/ or effect modifier, epidemiologic/ or (prognos* or confound* or "effect modif*" or predict* or score or factor* or regress* or hazard or associat* or "effect measure modif*" or subgroup* or correlat*).ab,ti. | 24484657 | Outcome string - title/abstract/MeSH/Emtree field search |
| 4 | 1 and 2 and 3 | 5650 |  |
| 5 | (line* or salvage* or rescue or prior).ab,ti. | 6209817 | Treatment line |
| 6 | (survival or respon* or remission or control or "time to next treatment" or progress* or outcome* or transform* or switch* or mortality or death).ab,ti. | 23957181 | Clinical outcomes |
| 7 | 4 and 5 and 6 | 2387 | Population + outcome + treatment line + clinical outcome strings |
| 8 | remove duplicates from 7 | 1831 | deduped |
| 9 | limit 8 to yr="2016 -Current" | 882 |  |
| 10 | limit 9 to english language | 865 |  |
| 11 | 10 not (commentary or editorial or letter or "case report*" or "case stud*").pt. | 856 |  |

**Appendix B – Quality assessment tool**

Risk of bias assessment of individual studies was performed using the quality in prognostic studies (QUIPS) tool [1].

| Variable | Bias Domains | | | | | |
| --- | --- | --- | --- | --- | --- | --- |
|  | 1. Study Participants | 2. Study Attrition | 3. Prognostic Factor Measurement | 4. Outcome Measurement | 5. Study Confounding | 6. Statistical Analysis and Reporting |
| Optimal study or characteristics of unbiased study | The study sample adequately represents the population of interest | The study data available (i.e., participants not lost to follow-up) adequately represent the study sample | The PF is measured in a similar way for all participants | The outcome of interest is measured in a similar way for all participants | Important potential confounding factors are appropriately accounted for | The statistical analysis is appropriate, and all primary outcomes are reported |
| Prompting items and considerations | a. Adequate participation in the study by eligible persons | a. Adequate response rate for study participants | a. A clear definition or description of the PF is provided | a. A clear definition of the outcome is provided | a. All important confounders are measured | a. Sufficient presentation of data to assess the adequacy of the analytic strategy |
|  | b. Description of the source population or population of interest | b. Description of attempts to collect information on participants who dropped out | b. Method of PF measurement is adequately valid and reliable | b. Method of outcome measurement used is adequately valid and reliable | b. Clear definitions of the important confounders measured are provided | b. Strategy for model building is appropriate and is based on a conceptual framework or mode |
|  | c. Description of the baseline study sample | c. Reasons for loss to follow-up are provided | c. Continuous variables are reported or appropriate cut points are used | c. The method and setting of outcome measurement is the same for all study participants | c. Measurement of all important confounders is adequately valid and reliable | c. The selected statistical model is adequate for the design of the study |
|  | d. Adequate description of the sampling frame and recruitment | d. Adequate description of participants lost to follow-up | d. The method and setting of measurement of PF is the same for all study participants |  | d. The method and setting of confounding measurement are the same for all study participants | d. There is no selective reporting of results |
|  | e. Adequate description of the period and place of recruitment | e. There are no important differences between participants who completed the study and those who did not | e. Adequate proportion of the study sample has complete data for the PF |  | e. Appropriate methods are used if imputation is used for missing confounder data |  |
|  | f. Adequate description of inclusion and exclusion criteria |  | f. Appropriate methods of imputation are used for missing PF data |  | f. Important potential confounders are accounted for in the study design |  |
|  |  |  |  |  | g. Important potential confounders are accounted for in the analysis |  |
| Ratings |  |  |  |  |  |  |
| High risk of bias | The relationship between the PF and outcome is very likely to be different for participants and eligible nonparticipants | The relationship between the PF and outcome is very likely to be different for completing and noncompleting participants | The measurement of the PF is very likely to be different for different levels of the outcome of interest | The measurement of the outcome is very likely to be different related to the baseline level of the PF | The observed effect of the PF on the outcome is very likely to be distorted by another factor related to PF and outcome | The reported results are very likely to be spurious or biased related to analysis or reporting |
| Moderate risk of bias | The relationship between the PF and outcome may be different for participants and eligible nonparticipants | The relationship between the PF and outcome may be different for completing and noncompleting participants | The measurement of the PF may be different for different levels of the outcome of interest | The measurement of the outcome may be different related to the baseline level of the PF | The observed effect of the PF on outcome may be distorted by another factor related to PF and outcome | The reported results may be spurious or biased related to analysis or reporting |
| Low risk of bias | The relationship between the PF and outcome is unlikely to be different for participants and eligible nonparticipants | The relationship between the PF and outcome is unlikely to be different for completing and noncompleting participants | The measurement of the PF is unlikely to be different for diverse levels of the outcome of interest | The measurement of the outcome is unlikely to be different related to the baseline level of the PF | The observed effect of the PF on outcome is unlikely to be distorted by another factor related to PF and outcome | The reported results are unlikely to be spurious or biased related to analysis or reporting |

PF, prognostic factor.

**Appendix C – List of variables**

| Categories | Variable | Included in the questionnaire |
| --- | --- | --- |
| Identified in SLR | | |
| Patient demographics and clinical characteristics | Age | Yes |
|  | Karnofsky score | No – used to augment ECOG |
|  | ECOG performance status | Yes |
|  | HCT-CI | No – treatment-specific variable |
| Disease and treatment characteristics | Chemosensitivity | Yes – revised to chemorefractory or chemoresistant |
|  | Prior lines of therapy | Yes |
|  | FLIPI score | Yes |
|  | Disease status at transplant | No – treatment-specific variable |
|  | Conditioning regimen | No – treatment-specific variable |
|  | GVHD grade | No – treatment-specific variable |
|  | Histology | Yes – revised to grade |
|  | Ann Arbor stage | Yes |
|  | Disease stage at diagnosis | No – similar to Ann Arbor stage |
|  | Extranodal involvement at HCT | No – treatment-specific variable |
|  | Nodal sites involved | Yes |
|  | PFS2 | No – outcome variable |
|  | POD24 | Yes |
|  | History of early treatment failure | No – captured by POD24 |
|  | Duration of last remission prior to allo-SCT | No – treatment-specific variable |
|  | Time between ASCT and relapse | No – treatment-specific variable |
|  | Treatment line for ASCT | No – treatment-specific variable |
|  | Histological transformation at relapse after ASCT | No – treatment-specific variable |
| Lab measures | Hemoglobin | Yes – revised to hemoglobin at relapse |
|  | LDH | Yes |
|  | Serum soluble interleukin 2-receptor level at third line | No – unavailable in the single-arm trial and RWD |
|  | SUVmax in PET/CT | Yes |
|  | TMTV | No – unavailable in the single-arm trial and RWD |
|  | Deauville score | Yes |
| Added by the study team based on expert suggestion | | |
|  | Serum beta-2 microglobulin | Yes |
|  | Bulky disease | Yes |
|  | Refractory to rituximab | Yes |
|  | Response to last LoT | Yes |
|  | Refractory to last LoT | Yes |
|  | Time of first relapse after diagnosis | Yes |
|  | Positive PET | Yes |
| Abbreviations: allo-SCT, allogeneic stem cell transplant; ASCT, autologous stem-cell transplant; ECOG, Eastern Cooperative Oncology Group; FLIPI, Follicular Lymphoma International Prognostic Index; GVHD, graft-versus-host disease; HCT-CI, hematopoietic cell transplant-comorbidity index; LDH, lactate dehydrogenase; LoT, line of therapy; PET/CT, positron emission tomography/computed tomography; PFS2, interval between frontline treatment and the second relapse; POD24, progression of disease within 2 years; RWD: real-world data; SUVmax: standardize uptake value maximum; TMTV, total metabolic tumor volume | | |

**Appendix D – Online questionnaire completed by the clinical experts**

Note: Questions 2-14 were related to DLBCL, which is not a topic for this manuscript, therefore only the questions related to FL are presented in this appendix.


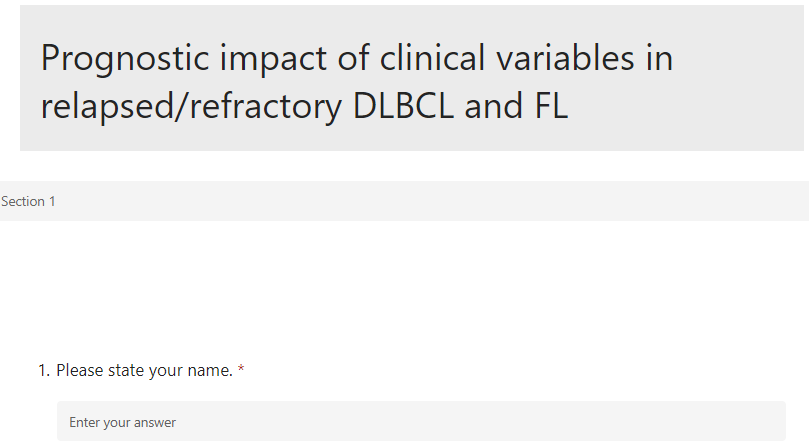


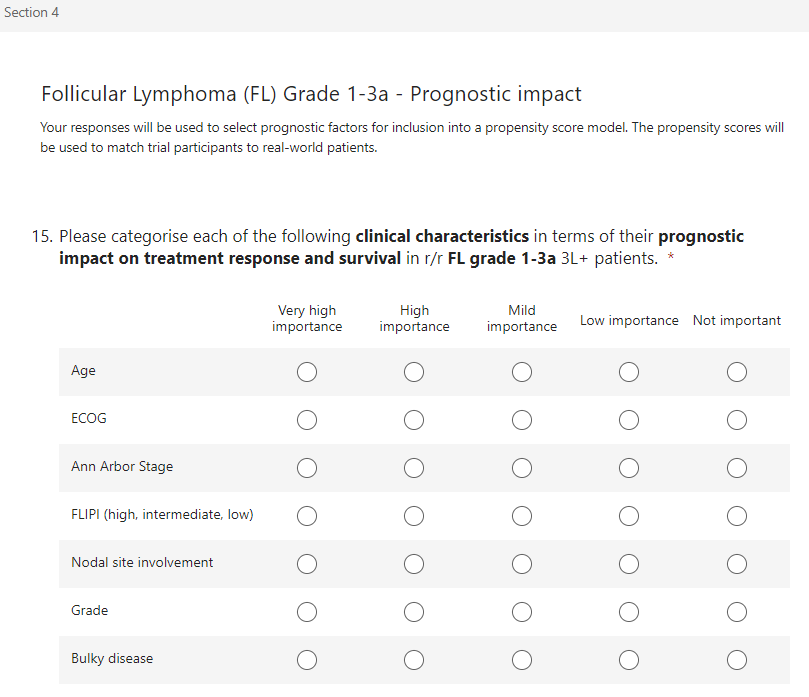


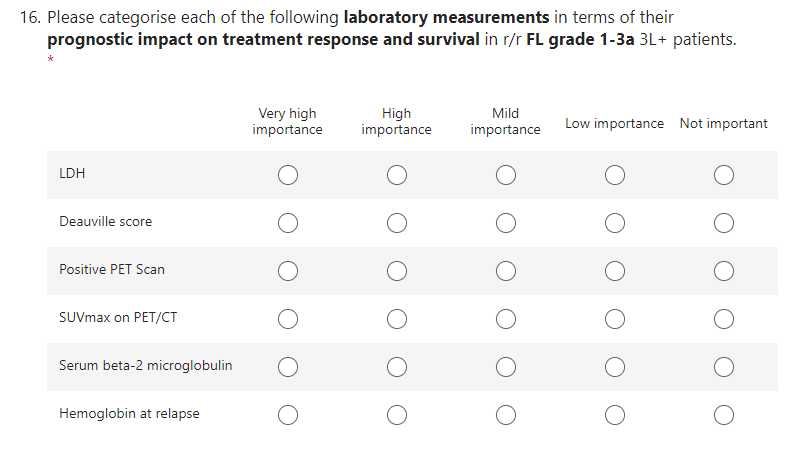


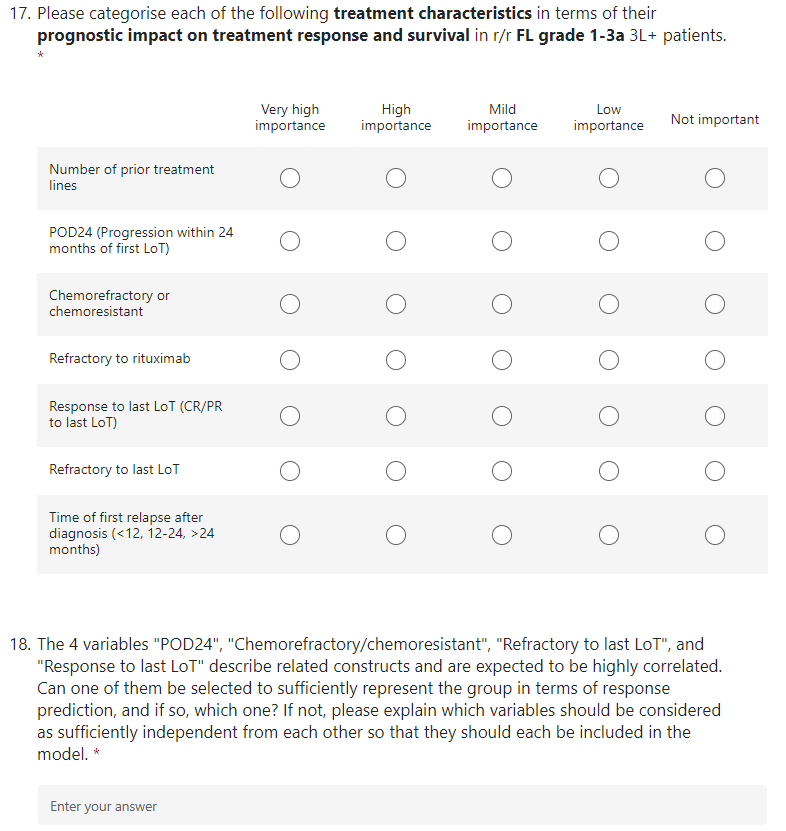


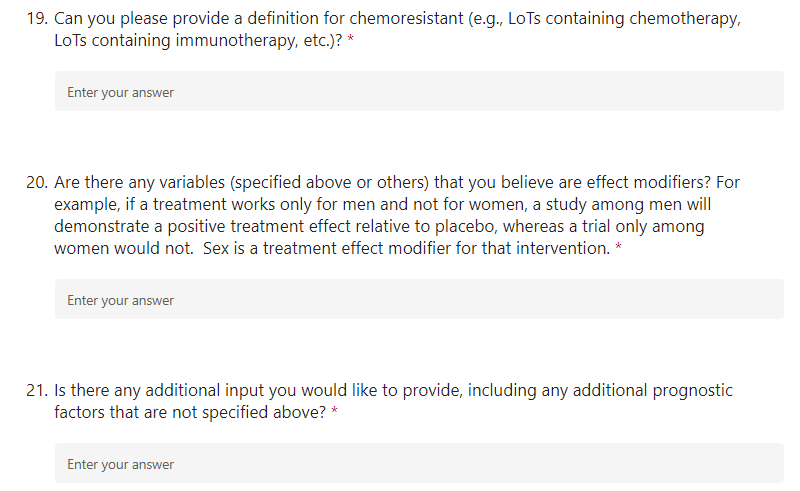


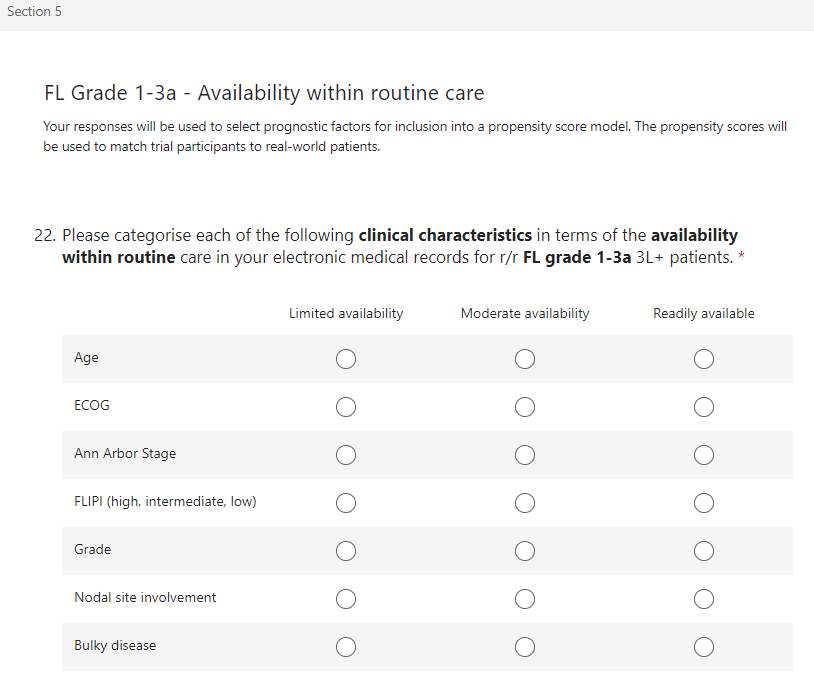


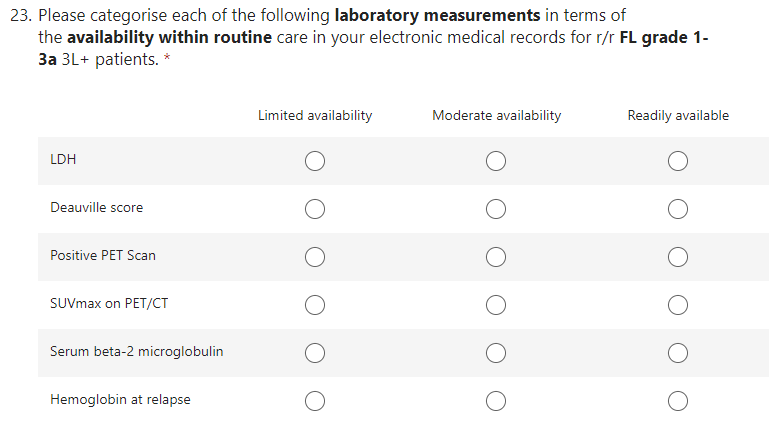


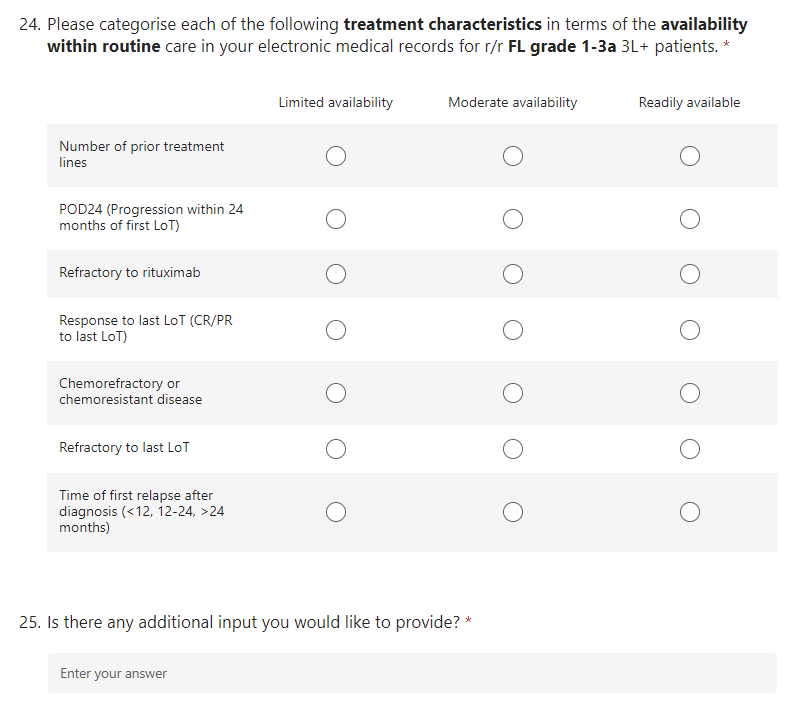


**Appendix E – Supplementary data**

Supplementary Table 1. Study characteristics

Supplementary Table 2. Patient demographics, clinical characteristics, disease characteristics, treatment characteristics, and laboratory measures that have statistically significant association with a clinical outcome

**Supplementary Table 1. Study characteristics**

| Author year  Study name; trial # | Study country | Study period | Median follow-up | N for FL | Intervention /comparator | Source of population | Population description | Analytical approach | Median line of prior therapy | Median age |
| --- | --- | --- | --- | --- | --- | --- | --- | --- | --- | --- |
| *Non-randomized trial* | | | | | | | | | | |
| Thieblemont 2021^a^[2]  ELARA; NCT03568461 | US, Australia, Austria, Belgium, France, Germany, Italy, Japan, Netherlands Norway, Spain, UK | November 12, 2018-March 29, 2021 | 17 months | 94 | Tisagenlecleucel | ELARA study | Adult patients with histologically confirmed r/r FL (grades 1-3A) after ≥2 lines of therapy or had relapsed after autologous stem cell transplant | Descriptive subgroup analysis was supported by MVA to identify factors predictive of worse outcomes | Number of prior lines of therapy  <5: 67^b^  ≥5: 27 | NR |
| Bartlett 2018[3]  NCT01849263 | Canada, Singapore, US | April 2013-April 2014 | 25.5 months (range, 11.3-33.1) | 40 | Ibrutinib | NCT01849263, clinical trial | Patients with confirmed grade 1, 2, or 3A FL recurring after 1 or more chemotherapy regimens | Univariate cox proportional hazards model for PFS and Wilcoxon rank sum test for correlation with best response | 3 (1-11)^b^ | 64 (46-82) |
| *Observational study* | | | | | | | | | | |
| Metzner 2021[4] | Germany | 1993-2020 | 140.4 months (range 6.0-327.6) | 76 | ASCT with TBI and CP in addition to ASCT with BEAM/ NA | Institutional data | Patients with FL | Univariable and Multivariable Cox proportional hazards including backward selection to develop a reduced model | 1L: ASCT with BEAM, CP and TBI: 2 (2-2)  2L: ASCT with BEAM: 3 (1-5)^b^  3/4L: ASCT with BEAM: 3 (2-5) | 1L: ASCT with BEAM, CP and TBI: 48 (25-60)  2L: ASCT with BEAM: 53 (31-69)  3/4L: ASCT with BEAM: 56 (53-65) |
| Montoro 2021[5] | Spain | January 2000 to December 2018 | 108 months (range 4–225) | 194 | AlloHSCT/ NA | GETH center | FL patients after Allo-SCT using HLA-matched sibling, unrelated, and haploidentical-related donors | A Cox proportional hazards model or the Fine and Gray method for competing events were used for MVA | Number of prior lines of therapy  2: 53 3: 58 >3: 80 | Overall: 50 (29-70)  MSD: 49 (29-67)  MUD: 50 (31-70)  Haploidentical: 55 (39-70) |
| Sakurai 2021[6] | Japan | 2001-2017 | 67 months (range, 1-201) after transplantation | 141 | AlloHSCT/ NA | Transplant registry unified management program database | Patients with FL relapsing after autologous transplantation | MVA of estimated risk factors for transplant outcomes were performed using the log-rank test, Cox proportional hazard regression analysis, and Fine-Gray proportional hazards model | 4 (1-21) ^b^ | 53 (32-76) |
| Szlauer‐Stefańska 2021^a^[7] | Poland | 2000-2019 | 48 months (range 1‐235) | 66 | AutoHSCT | 3 centres from the Polish lymphoma research group | R/R FL and a history of rituximab treatment | NR | 2 (1-7) ^b^ | 51 (22-73) |
| Mozas 2020^a^[8] | Spain | 2002-2018 | 117.6 months (range, 33.6-214.8) | 89 | NR | Two tertiary hospitals | Patients with FL who had relapsed after or were refractory to at least two lines of treatment | NR | Patients who had relapsed after or were refractory to at least two lines of treatment. | NR |
| Fuji 2020[9] | Japan | January 2007-June 2017 | 89.7 months (28.5-134.4) (surviving patients) | 41 | Third-line chemotherapy (second-line salvage therapy), and RTX-containing chemotherapy as first-line chemotherapy | 8 cancer institutes | Patients with multiple RR FL | The OS and PFS rates were computed from the survival curves estimated by the Kaplan–Meier method, and groups were compared using the log-rank test. For all analyses, a two-sided P < 0.05 was considered statistically significant | Patients with relapsed/refractory follicular lymphoma after third-line chemotherapy | 59 (38-70) |
| Sesques 2020[10] | France | 2000-2014 | 105.6 months (range, 27.6-291.6) | 95 | ASCT | Medical records collected from 4 hematologic departments: Léon Bérard center (Lyon), University Hospitals of Lyon, Nantes and Dijon | FL grade 1, 2, or 3A patients older than 18 years | MVA using Cox proportional hazards model. A level of significance of 0.05 was considered statistically significant | 2 (2-6) | 57 (35-71) |
| Sesques 2017^a^[11] |  |  |  |  |  |  |  |  |  |  |
| Khouri 2018^a^[12] | US | 1999-2017 | 98 months (range, 3-208) months | 98 | Non-Myeloablative Allo-SCT | 3 consecutive trials between 1999-2017 | Relapsed/chemosensitive FL | MVA | 3 (2-9) | 53 (29-71) |
| Smith 2018[13]  CIBMTR 2002-2014^c^ | US | 2002-2014 | 69-73 months | 440 | Auto-HCT | CIBMTR database | FL patients experiencing early treatment failure | Cox proportional hazards regression. Covariates with a p<0.05 were considered significant | Auto HCT: 2 (1-6)^b^  MSD: 3 (1-9)^b^  MUD: 3 (1-8)^b^ | Auto HCT: 56 (23-79)  MSD: 52 (29-68)  MUD: 53 (21-74) |
| Sureda 2018[14]  EBMT and CIBMTR 2001-2011^c^ | International | January 2001-December 2011 | 55 months (range, 3-160) | 1567 | Allo-SCT | EBMT and CIBMTR | Recipients of allo-HCT with FL | MVA using Cox proportional hazards model | Number of prior chemotherapy regimens:  1-2: 291^b^  3-4: 397  ≥5: 172 | Overall population: 51 (21-74)  AlloHSCT: CIBMTR: 50 (21-74)  AlloHSCT: EBMT: 51 (24-72) |
| Sureda 2016^a^[15] |  |  |  |  |  |  |  |  |  |  |
| Robinson 2016[16]  EBMT 1998-2012^c^ | Europe | 1998-2012 | 58.8 months (range 3–159) | 183 | RIC-allo-SCT | EBMT | Patients with FL relapsing after an autoSCT | Multivariate Cox proportional hazards regression analysis | 4 (3-10) | At diagnosis: 45 (21-69)  At transplant: 52 (24-73) |

^a^Conference abstract

^b^This SLR included FL patients who failed at least 2 lines of prior therapy. This symbol indicates that the study had a mix population involving some patients who received only 1 line of prior therapy, but had 50%+ received at least 2 lines of prior therapy or had a median/mean of at least 2 lines of prior therapy

^c^Some studies involved patients from the same data source, and they were presented individually since potentially different sub-population and analysis were involved. The included population could be overlapping among these studies.

Abbreviations: Allo-HSCT, allogeneic hematopoietic stem cell transplantation; allo-HCT, allogeneic hematopoietic cell transplantation; ASCT, autologous stem cell transplantation; auto-HCT, autologous hematopoietic cell transplantation; CP, cyclophosphamide; CIBMTR, Center for International Blood and Marrow Transplant Research; EBMT, The European Blood and Marrow Transplantation; FL, follicular lymphoma; GETH: Grupo Español de Trasplante Hematopoyético y Terapia Celular; HLA, Human Leukocyte Antigen; JSHCT, Japan Society for Hematopoietic Cell Transplantation; JDCHCT, Japanese Data Center for Hematopoietic Cell Transplantation; MVA, multivariate analysis; MSD, matched sibling donor; MUD, matched unrelated donor; NMA, non-myeloablative; NR, not reported; NA, not applicable; OS, overall survival; PFS, progression free survival; PLRG, Polish Lymphoma Research Group; RIC-allo-SCT, reduced intensity allogeneic stem cell transplantation; RTX, rituximab; RCT, randomized clinical trials; r/r, relapsed/refractory; TRUMP, Transplant Registry Unified Management Program; TBI, total body irradiation; US, United States; UK, United Kingdom.

**Supplementary Table 2.** **Patient demographics, clinical characteristics, disease characteristics, treatment characteristics, and laboratory measures that have statistically significant association with a clinical outcome**

| Variable | Clinical outcomes | Author Year | Parameter (vs reference)  - category with favorable outcome labeled in green | N for parameter /reference | Effect estimates  (95% CI) | P-value | Supportive evidence | Statistical method | Multivariate /univariate analysis | Study design | Publication type |
| --- | --- | --- | --- | --- | --- | --- | --- | --- | --- | --- | --- |
| ***Age: Older age was associated with worse outcomes in 3 studies*** | | | | | | | | | | | |
| Age | OS | Sureda 2018^a^[14]  EBMT and CIBMTR 2001-2011^b^ | Per year of age  *(continuous)* | 1523 total | RR: 1.03 (1.02, 1.04) | <0.0001 | “Age at transplantation was adverse prognostic factors (for OS)” | Cox proportional hazards analysis | Multivariate | Observational | Journal article |
|  |  | Robinson 2016[16]  EBMT 1998-2012^b^ | ≥45 years (vs <45 years) | 183 total | RR: 1.7 (1.0, 2.7) | 0.04 | “Age over 45 at the time of allogeneic SCT was the only factors associated with a significantly worse…OS in a multivariate analysis” | Cox proportional hazards analysis | Multivariate | Observational | Journal article |
|  | PFS | Montoro 2021[5] | >50 years (vs ≤50 years) | 194 total | HR: 2.3 (1.5, 3.7) | 0.001 | “In multivariable analysis, age >50 years (HR 2.3, 95% CI, 1.5–3.7, P < 0.001), was independently associated with a lower PFS” | Cox proportional hazards analysis | Multivariate | Observational | Journal article |
|  |  | Robinson 2016[16]  EBMT 1998-2012^b^ | ≥45 years (vs <45 years) | 183 total | RR: 1.8 (1.1, 2.9) | 0.02 | “Age over 45…at the time of allogeneic SCT were the only factors associated with a significantly worse PFS…in a multivariate analysis” | Cox proportional hazards analysis | Multivariate | Observational | Journal article |
|  | NRM | Montoro 2021[5] | >50 years (vs ≤50 years) | 194 total | HR: 2.3 (1.3, 4.0) | 0.002 | “Variable associated with increased NRM was age at transplant >50 (hazard ratio (HR) 2.3, 95% CI, 1.3–4, P=0.002)” | Cox proportional hazards analysis | Multivariate | Observational | Journal article |
|  |  | Sureda 2018^a^[14]  EBMT and CIBMTR 2001-2011^b^ | Per year of age  *(continuous)* | 1567 total | RR: 1.04 (1.02, 1.05) | <0.0001 | “Multivariate analysis indicated that NRM was significantly affected by age (HR 1.04, 1.02-1.05, p<0.0001)” | Cox proportional hazards analysis | Multivariate | Observational | Journal article |
|  |  | Robinson 2016[16]  EBMT 1998-2012^b^ | ≥45 years (vs <45 years) | 183 total | RR: 2.1 (1.2, 3.6) | 0.01 | “Multivariate analysis identified age >45…as being associated with higher risk of NRM” | Cox proportional hazards analysis | Multivariate | Observational | Journal article |
|  | TRM | Sureda 2018^a^[14]  EBMT and CIBMTR 2001-2011^b^ | Per year of age  *(continuous)* | 1523 total | RR: 1.04 (1.02, 1.05) | < 0.0001 | “Age, … were associated to a higher TRM in the multivariate analysis” | Cox proportional hazards analysis | Multivariate | Observational | Journal article |
|  | Progression/  relapse | Robinson 2016[16]  EBMT 1998-2012^b^ | ≥45 years (vs <45 years) | 183 total | RR: 2.1 (1.2, 3.5) | 0.009 | “Age >45 was the only factor associated with a significantly higher relapse rate in the multivariate analysis” | Cox proportional hazards analysis | Multivariate | Observational | Journal article |
| ***Karnofsky score (KPS): Lower KPS (<80 or < 90) was an independent adverse prognostic factor in 2 studies*** | | | | | | | | | | | |
| KPS | OS | Sureda 2018^a^[14]  EBMT and CIBMTR 2001-2011^b^ | <80 (vs ≥80) | 54/986 | RR: 2.23 (1.52, 3.25) | <0.0001 | “Poor PS was independent adverse prognostic factor” | Cox proportional hazards analysis | Multivariate | Observational | Journal article |
|  | PFS |  | <80 (vs ≥80) | 54/986 | RR: 1.78 (1.23, 2.58) | 0.002 | “Inadequate KPS was independent adverse prognostic factor” | Cox proportional hazards analysis | Multivariate | Observational | Journal article |
|  | NRM |  | <80 (vs ≥80) | 1567 total | HR: 2.05 (1.32, 3.19) | 0.0014 | “NRM was significantly affected by KPS <80” | Cox proportional hazards analysis | Multivariate | Observational | Journal article |
|  |  | Smith 2018^a^[13]  CIBMTR 2002-2014^b^ | <90 (vs ≥90) | 119/282 | RR: 2.09 (1.23, 3.57) | 0.007 | “Non-relapse mortality is significantly associated with KPS <90%” | Cox proportional hazards analysis | Multivariate | Observational | Journal article |
|  | TRM | Sureda 2018^a^[14]  EBMT and CIBMTR 2001-2011^b^ | <80 (vs ≥80) | 54/986 | RR: 2.05 (1.32, 3.19) | 0.001 | “Low KPS was associated to a higher TRM” | Cox proportional hazards analysis | Multivariate | Observational | Journal article |
| ***Patient clinical characteristics reported in 1 study*** | | | | | | | | | | | |
| ECOG performance status | OS | Sakurai 2021^a^[6] | 2-4 (vs 0-1) | 141 total | HR: 4.17 (1.89, 9.09) | <0.001 | “Poor performance status was associated with lower OS” | NR | Multivariate | Observational | Journal article |
|  | PFS | Sakurai 2021^a^[6] | 2-4 (vs 0-1) | 141 total | HR: 3.85 (1.69, 9.09) | 0.001 | “Poor performance status was associated with lower PFS” | NR | Multivariate | Observational | Journal article |
| HCT-CI | PFS | Montoro 2021[5] | High (vs Low) | 194 total | HR: 1.8 (1.1, 2.9) | 0.02 | “High HCT-CI was independently associated with a lower PFS” | Cox proportional hazards analysis | Multivariate | Observational | Journal article |
|  | NRM | Montoro 2021[5] | High (vs Low) | 194 total | HR: 1.8 (1.1, 3.2) | 0.03 | “Variables associated with increased NRM was high HCT-CI” | Cox proportional hazards analysis | Multivariate | Observational | Journal article |
| ***Chemosensitivity: Chemorefractory/chemoresistant disease was associated with worse outcomes in 3 studies*** | | | | | | | | | | | |
| Chemo-sensitivity | OS | Sureda 2018^a^[14]  EBMT and CIBMTR 2001-2011^b^ | Chemoresistant (vs chemosensitive)  *[Chemosensitive: at least partial remission was achieved following the last course of chemotherapy]* | 253/1158 | RR: 1.59 (1.30, 1.95) | <0.0001 | “Chemorefractory disease was adverse prognostic factor” | Cox proportional hazards analysis | Multivariate | Observational | Journal article |
|  |  | Robinson 2016[16]  EBMT 1998-2012^b^ | Chemosensitive (vs chemorefractory)  *[Chemosensitive: at least partial remission was achieved following the last course of chemotherapy]* | 183 total | RR: 0.38 (0.2, 0.7) | 0.001 | “Chemorefractory disease at the time of allogeneic SCT was associated with a significantly worse OS” | Cox proportional hazards analysis | Multivariate | Observational | Journal article |
|  | PFS | Sureda 2018^a^[14]  EBMT and CIBMTR 2001-2011^b^ | Chemoresistant (vs chemosensitive) | 253/1158 | RR: 1.54 (1.28, 1.86) | <0.0001 | “Chemorefractory disease was adverse prognostic factor” | Cox proportional hazards analysis | Multivariate | Observational | Journal article |
|  |  | Robinson 2016[16]  EBMT^b^ | Chemosensitive (vs chemorefractory) | 183 total | RR: 0.42 (0.2, 0.8) | 0.03 | “Chemorefractory disease at the time of allogeneic SCT was associated with a significantly worse PFS” | Cox proportional hazards analysis | Multivariate | Observational | Journal article |
|  | Non-relapse mortality | Sureda 2018^a^[14]  EBMT and CIBMTR 2001-2011^b^ | Chemoresistant (vs chemosensitive) | 1567 total | HR: 1.61 (1.28, 2.03) | <0.0001 | “Non-relapse mortality was significantly affected by chemoresistant disease” | Cox proportional hazards analysis | Multivariate | Observational | Journal article |
|  |  | Robinson 2016[16]  EBMT 1998-2012^b^ | Chemosensitive (vs chemorefractory) | 183 total | RR: 0.5 (0.2, 1.0) | 0.05 | “Chemorefractory disease as being associated with a higher risk of non-relapse mortality” | Cox proportional hazards analysis | Multivariate | Observational | Journal article |
|  | Transplant Related Mortality | Sureda 2018^a^[14]  EBMT and CIBMTR 2001-2011^b^ | Chemoresistant (vs chemosensitive) | 253/1158 | RR: 1.61 (1.28, 2.03) | <0.0001 | “Chemoresistant disease was associated to a higher Transplant Related Mortality” | Cox proportional hazards analysis | Multivariate | Observational | Journal article |
|  | Progression/ relapse | Sureda 2018^a^[14]  EBMT and CIBMTR 2001-2011^b^ | Chemoresistant (vs chemosensitive) | 253/1158 | RR: 1.46 (1.07, 1.97) | 0.015 | “Chemorefractory disease was  associated to a significantly higher relapse rate after the procedure” | Cox proportional hazards analysis | Multivariate | Observational | Journal article |
|  |  | Robinson 2016[16]  EBMT 1998-2012^b^ | Chemosensitive (vs chemorefractory) | 183 total | RR: 0.2 (0.1, 0.5) | 0.001 | “Chemorefractoriness at RICalloSCT was the only factor associated with a significantly higher relapse rate in the multivariate analysis” | Cox proportional hazards analysis | Multivariate | Observational | Journal article |
|  | ORR | Bartlett 2018^a^[3] | Rituximab-sensitive disease (vs rituximab-refractory disease)  *[Definition not reported]* | 19/18 | NR | 0.04 | “Response rates were higher among patients with rituximab-sensitive disease (52.6%) compared with those who had rituximab-refractory disease (16.7%)” | Cox proportional hazards analysis | Univariate | Non-randomized trial | Journal article |
| ***Prior lines of therapy/treatment line: Higher number of prior lines of therapy/treatment line was associated with worse outcomes in 2 studies*** | | | | | | | | | | | |
| Prior lines of therapy | OS | Sureda 2018^a^[14] | 3-4 (vs 1-2) | 390/285 | RR: 1.48 (1.11, 1.97) | 0.007 | “Chemotherapy burden before transplantation was adverse prognostic factor” | Cox proportional hazards analysis | Multivariate | Observational | Journal article |
|  |  |  | ≥5 (vs 1-2) | 165/285 | RR: 2.41 (1.77, 3.30) | <0.0001 |  |  |  |  |  |
|  |  |  | Missing (vs 1-2) | 683/285 | RR: 1.73 (1.33, 2.26) | <0.0001 |  |  |  |  |  |
|  | PFS | Montoro 2021[5] | >3 (vs ≤3) | 194 total | HR: 1.6 (1.0-2.6) | 0.03 | “>3 prior lines of therapy (HR 1.6, 95% CI, 1–2.6, = P 0.03) was independently associated with a lower PFS” | Cox proportional hazards analysis | Multivariate | Observational | Journal article |
|  |  | Sureda 2018^a^[14] | 3-4 (vs 1-2) | 390/285 | RR: 1.33 (1.04, 1.70) | 0.022 | “Number of prior lines of therapy was independent adverse prognostic factors” | Cox proportional hazards analysis | Multivariate | Observational | Journal article |
|  |  |  | ≥5 (vs 1-2) | 165/285 | RR: 1.93 (1.46, 2.55) | <0.0001 |  |  |  |  |  |
|  |  |  | Missing (vs 1-2) | 683/285 | RR: 1.56 (1.24, 1.95) | 0.0001 |  |  |  |  |  |
|  | NRM | Montoro 2021[5] | >3 (vs ≤3) | 194 total | HR: 1.8 (1.1, 3.0) | 0.01 | “Variables associated with increased NRM was: >3 prior therapy lines” | Cox proportional hazards analysis | Multivariate | Observational | Journal article |
|  |  | Sureda 2018^a^[14] | ≥5 (vs 3-4) | 1567 total | HR: 1.62 (1.20, 2.19) | 0.0015 | “NRM was significantly affected by ≥5 lines of prior CT (vs 3-4)” | Cox proportional hazards analysis | Multivariate | Observational | Journal article |
|  | TRM | Sureda 2018^a^[14] | 3-4 (vs 1-2) | 390/285 | RR: 1.56 (1.12, 2.17) | 0.008 | “Heavily pre-treated patients were associated to a higher TRM in the multivariate analysis” | Cox proportional hazards analysis | Multivariate | Observational | Journal article |
|  |  |  | ≥5 (vs 1-2) | 165/285 | RR: 2.53 (1.77, 3.62) | <0.0001 |  |  |  |  |  |
|  |  |  | Missing (vs 1-2) | 683/285 | RR: 1.84 (1.35, 2.51) | 0.0001 |  |  |  |  |  |
| ***FLIPI score: High risk FLIPI score was associated with worse outcomes in 2 studies*** | | | | | | | | | | | |
| FLIPI score | OS | Metzner 2021^a^[4] | High-risk (vs low-risk)  *[At salvage chemotherapy]* | 26/26 | HR: 3.75 (1.26, 11.19) | 0.018 | Data not interpreted in text | Cox proportional hazards analysis | Multivariate | Observational | Journal article |
|  | PFS | Sesques 2020[10] | High-risk (vs low-risk/Intermediate)  *[At time of relapse after ASCT]* | 95 total | HR: 2.469 (1.104, 5.521) | 0.028 | “FLIPI score was integrated as a single parameter, only the FLIPI score at relapse was associated with PFS“ | Cox proportional hazards analysis | Multivariate | Observational | Journal article |
| ***Disease status at transplant: Not achieving CR/PR at transplant was associated with worse outcomes in 2 studies*** | | | | | | | | | | | |
| Disease status at transplant | PFS | Montoro 2021[5] | No complete remission (vs complete remission) | 194 total | HR: 1.7 (1.1, 2.7) | 0.02 | “Not achieving complete remission (CR) prior to allo-HSCT was independently associated with a lower PFS” | Cox proportional hazards model | Multivariate | Observational | Journal article (Letter to Editor) |
|  |  | Sakurai 2021^a^[6] | Others (vs CR/PR) | 141 total | HR: 1.73 (1.06, 2.82) | 0.03 | “Advanced disease status at transplantation (CR/PR vs. others) was associated with lower PFS” | NR | Multivariate | Observational | Journal article |
|  | Progression/ relapse | Sakurai 2021^a^[6] | Others (vs CR/PR) | 141 total | HR: 3.57 (1.17, 10.88) | 0.03 | “Advanced disease status at transplantation (CR/PR vs. others) was associated with higher relapse/progression” | NR | Multivariate | Observational | Journal article |
| ***Conditioning regimen: The use of myeloablative conditioning regimen was an independent adverse prognostic factor, compared with reduced intensity conditioning/non-myeloablative conditioning regimen in 2 studies*** | | | | | | | | | | | |
| Conditioning regimen | OS | Sureda 2018^a^[14] | MAC (vs RIC/NMA) | 355/1168 | RR: 1.42 (1.16, 1.73) | 0.0006 | “MAC protocols was independent adverse prognostic factors” | Cox proportional hazards analysis | Multivariate | Observational | Journal article |
|  | PFS | Sureda 2018^a^[14] | MAC (vs RIC/NMA) | 355/1168 | RR: 1.36 (1.14, 1.63) | 0.0008 |  | Cox proportional hazards analysis | Multivariate | Observational | Journal article |
|  |  | Sakurai 2021^a^[6] | Reduced intensity (vs myeloablative) | 141 total | HR: 0.58 (0.35, 0.97) | 0.04 | “Regarding types of conditioning, RIC regimen provided a significantly  better PFS than MAC” | NR | Multivariate | Observational | Journal article |
|  | TRM | Sureda  2018^a^[14] | MAC (vs RIC/NMA) | 355/1168 | RR: 1.49 (1.18, 1.87) | 0.0007 | “The use of MAC regimens was associated to a higher TRM” | Cox proportional hazards analysis | Multivariate | Observational | Journal article |
| ***GVHD grade: Higher GVHD grade was associated with worse outcomes in 2 studies*** | | | | | | | | | | | |
| GVHD grade | OS | Khouri 2018[12] | Acute II-IV (vs I-II) | 22/76 | HR: 8.61 (2.99, 24.83) | <0.001 | “Acute II-IV GVHD [HR 8.61 (2.99, 24.83) p<0.001] associated with inferior OS” | NR | Multivariate | Observational | Conference abstract |
|  | NRM | Montoro 2021[5] | III-IV (vs I-II) | 194 total | HR: 4.3 (2.5, 7.5) | <0.001 | “Variables associated with increased NRM was grade III–IV aGVHD” | Cox proportional hazards analysis | Multivariate | Observational | Journal article |
| ***Disease and treatment characteristics reported in 1 study*** | | | | | | | | | | | |
| Histology | OS | Sureda 2018^a^[14] | Grade 3 (vs Grade 1) | 308/444 | RR: 1.44 (1.13, 1.83) | 0.003 | “Grade 3 histology was independent adverse prognostic factor” | Cox proportional hazards analysis | Multivariate | Observational | Journal article |
|  |  | Sureda 2018^a^[14] | Missing (vs Grade 1) | 388/444 | RR: 1.29 (1.01, 1.63) | 0.039 | Data not interpreted in text | Cox proportional hazards analysis | Multivariate | Observational | Journal article |
|  | PFS | Sureda 2018^a^[14] | Grade 3 (vs Grade 1) | 308/444 | RR: 1.42 (1.15, 1.76) | 0.001 | “Grade 3 histology was independent adverse prognostic factor” | Cox proportional hazards analysis | Multivariate | Observational | Journal article |
|  | Progression/  relapse | Sureda 2018^a^[14] | Grade 3 (vs Grade 1) | 308/444 | RR: 1.63 (1.16, 2.28) | 0.004 | “grade 3 histology were associated to a significantly higher relapse rate after the procedure” | Cox proportional hazards analysis | Multivariate | Observational | Journal article |
| Ann Arbor stage | OS | Sesques 2020[10] | I/II (vs III/IV) | 95 total | HR: 0.344 (0.123, 0.965) | 0.043 | “Disseminated disease (stage III/IV) was adversely associated with OS” | Cox proportional hazards analysis | Multivariate | Observational | Journal article |
| Disease Stage at diagnosis | Progression/  relapse | Smith 2018^a^[13] | III/IV (vs I/II) | 67/354 | RR: 1.66 (1.08, 2.56) | 0.02 | “Stage III/IV at diagnosis was an independent predictor for the risk of relapse/progression (RR=1.66)” | Cox proportional hazards analysis | Multivariate | Observational | Journal article |
| Extra nodal involvement at HCT | OS | Smith 2018^a^[13] | Yes (vs No) | 103/327 | RR: 1.93 (1.37, 2.72) | 0.0002 | “Significantly associated with mortality risk included extra nodal involvement at HCT” | Cox proportional hazards analysis | Multivariate | Observational | Journal article |
|  |  |  | Missing (vs No) | 10/327 | RR: 3.76 (1.73, 8.18) | 0.0008 | Data not interpreted in text | Cox proportional hazards analysis | Multivariate | Observational | Journal article |
| Nodal sites involved | OS | Sesques 2020[10] | ≥4 (vs <4) | 95 total | HR: 2.646 (1.034, 6.772) | 0.042 | “In multivariate analysis, in the second model, when all the FLIPI score, items were analyzed disseminated disease (≥4 involved nodal sites) was negatively associated with OS” | Cox proportional hazards analysis | Multivariate | Observational | Journal article |
| PFS2 | OS | Mozas 2020[8] | >5Y or 2-5Y (vs <2Y) | 19/29/41 | NR | 0.0016 | “SFR2 (survival from second relapse, defined as interval between the second relapse and last follow-up) was significantly different among patients with a PFS2 < 2y, 2-5y, and > 5y (39 vs. 67 vs. 88% at 5 years, P = 0.0016)” | NR | Not specified | Observational | Conference abstract |
|  | Complete response after 3rd line treatment | Mozas 2020[8] | >5Y or 2-5Y (vs <2Y) | 19/29/41 | NR | < 0.001 | “A shorter PFS2 was predictive of a lower likelihood of achieving a complete response after 3rd line treatment (32 vs. 44 vs. 100% for patients with a PFS2 < 2y, 2-5y, and > 5y, respectively, P < 0.001)” | NR | Not specified | Observational | Conference abstract |
| POD24 | PFS | Thieblemont 2021^a^ [2] | Yes (vs No) | 61/33 | HR: 2.34 (1.02, 5.34) | NR | “POD24 was associated with shorter PFS” | NR | Multivariate | Non-randomized trial | Conference abstract |
| History of early treatment failure (ETF) | OS | Szlauer‐Stefańska 2021^a^[7] | Yes (vs No) | 66 total | NR | 0.04 | “Probability of OS at 5 years was decreased for patients with prior ETF (less than 24 months)” | NR | Univariate | Observational | Conference abstract |
| Duration of last remission prior to alloSCT | OS | Khouri 2018[12] | <1 Year (vs >1 Year) | 60/38 | HR: 6.48 (1.28, 32.69) | 0.024 | “Duration of last remission prior to alloSCT (<1year) was associated with inferior OS” | NR | Multivariate | Observational | Conference abstract |
| Time between ASCT and relapse | OS | Sesques 2020[10] | >2 years (vs <2 years) | 95 total | HR: 0.2 (0.04, 1.00) | 0.05 | “Early relapse after ASCT (<2 years) was associated with OS” | Cox proportional hazards model | Multivariate | Observational | Journal article |
| Treatment line for ASCT | OS | Metzner 2021^a^[4] | 3rd/4th (vs 1st) | 8/20 | RR: 6.7 (1.6, 28.01) | 0.009 | Data not interpreted in text | Cox proportional hazards analysis | Multivariate | Observational | Journal article |
| Histological transformat--ion at relapse after ASCT | OS | Sesques 2020[10] | Yes (vs No) | 95 total | HR: 3.730 (1.364, 10.199) | 0.01 | “In multivariate analysis, in the second model, when all the FLIPI score items were analyzed, HT was associated with OS” | Cox proportional hazards analysis | Multivariate | Observational | Journal article |
| ***Lab measures reported in 1 study*** | | | | | | | | | | | |
| Hemoglobin | OS | Sesques 2020[10] | ≤12 g/dL (vs >12 g/dL) | 95 total | HR: 3.411 (1.286, 9.049) | 0.014 | Data not interpreted in text | Cox proportional hazards analysis | Multivariate | Observational | Journal article |
|  | PFS | Sesques 2020[10] | ≤12 g/dL (vs >12 g/dL) | 95 total | HR: 2.438 (1.223, 4.863) | 0.011 | Data not interpreted in text | Cox proportional hazards analysis | Multivariate | Observational | Journal article |
| LDH | OS | Smith 2018^a^[13] | High (vs Normal) | 142/240 | RR: 1.57 (1.13, 2.19) | 0.008 | “Factors significantly associated with mortality risk included elevated LDH” | Cox proportional hazards analysis | Multivariate | Observational | Journal article |
|  | NRM | Smith 2018^a^[13] | High (vs Normal) | 137/238 | RR: 1.92 (1.13, 3.28) | 0.02 | “Factors significantly associated with the risk of NRM include elevated LDH” | Cox proportional hazards analysis | Multivariate | Observational | Journal article |
| sIL2R at third-line | PFS | Fuji 2020[9] | >1080 IU/mL (vs  ≤ 1080 IU/mL) | 17/20 | NR | 0.036 | “When grouped according to the serum sIL-2R level (median, 1080 IU/mL), the median PFS and probability of PFS at 2 years were 3.33 years (95% CI, 0.58–NA) and 65.5% (95% CI, 33.6–84.9), respectively, in patients with a serum sIL-2R level above the median, and 0.92 years (95% CI, 0.26– 1.61) and 20.5% (95% CI, 5.4–42.5), respectively, in patients with a serum sIL-2R level below or equal to the median (P = 0.036)” | Log rank test | Univariate | Observational | Journal article |
| SUVmax in PET/CT | PFS | Bartlett 2018^a^[3] | SUVmax at cycle 1 day 8  *(continuous)* | 9/11 | NR | 0.03 | “SUVmax as a continuous measure at cycle 1 day 8 correlated with PFS  (P = .03)” | Cox proportional hazards analysis | Univariate | Non-randomized trial | Journal article |
|  |  | Bartlett 2018^a^[3] | At cycle 1 day 8 PET/CT;  ≥13.78 (vs <13.78) | 9/11 | NR | 0.0004 | “PFS curve for patients whose day 8 SUVmax was >=13.78 vs <13.78 (P = .0004)” | Cox proportional hazards analysis | Univariate | Non-randomized trial | Journal article |
|  | ORR | Bartlett 2018^a^[3] | SUVmax at cycle 1 day 8  *(continuous)* | 9/11 | NR | 0.03 | “SUVmax as a continuous measure at cycle 1 day 8 correlated with response  (P =.03)” | Cox proportional hazards analysis | Univariate | Non-randomized trial | Journal article |
| Total metabolic tumor volume | PFS | Thieblemont 2021^a^[2] | High (>510 cm^3, vs Low) | 20/72 | HR: 2.53 (1.14, 5.65) | NR | “High TMTV was associated with shorter PFS” | NR | Multivariate | Non-randomized trial | Conference abstract |
| Deauville score | OS | Szlauer‐Stefańska 2021[7] | ≥3 (vs <3) | 66 total | NR | 0.02 | “Probability of OS at 5 years was decreased for patients with Deauville ≥3 (83.3% % vs 97.7%, p = 0.02)” | NR | Univariate | Observational | Conference abstract |

^a^This SLR included FL patients who failed at least 2 lines of prior therapy. This symbol indicates that the study had a mix population involving some patients who received only 1 line of prior therapy, but had 50%+ received at least 2 lines of prior therapy or had a median/mean of at least 2 lines of prior therapy

^b^Studies from the same source of population

Abbreviation: Allo-SCT, allogenic stem cell transplantation; CI, Confidence interval; CIBMTR, Center for International Bone Marrow Transplantation Research; CR, complete response; CT, chemotherapy; CT, computed tomography; ECOG, Eastern Cooperative Oncology Group; EBMT, European Society for Blood and Marrow Transplantation; ETF, early treatment failure; FLIPI, Follicular Lymphoma International Prognostic Index score; GVHD, graft versus host disease; HCT-CI, hematopoietic cell transplant-comorbidity index; HR, hazard ratio; HT, histologic transformation; KPS, Karnofsky performance status; LDH, lactate dehydrogenase; MAC, myeloablative conditioning regimen; Max, maximum; NMA, non-myeloablative; NR, not reported; NRM, non-relapse mortality; ORR, objective response rate; OS, overall survival; PFS, progression free survival; POD24, progression of disease within 24 months from the start of the first-line treatment; PR, partial response; RIC, reduced intensity conditioning; RR, risk ratio; sIL2R, serum soluble interleukin 2-receptor level; SUV, standardized uptake value; TMTV, total metabolic tumor volume; TRM, transplant related mortality; Y, year

**References for supplemental appendix**

1. Hayden JA, van der Windt DA, Cartwright JL, Cote P, Bombardier C (2013) Assessing bias in studies of prognostic factors. Ann Intern Med 158 (4):280-286. doi:10.7326/0003-4819-158-4-201302190-00009

2. Thieblemont C, Dickinson M, Martinez-Lopez J, Kolstad A, Butler JP, Ghosh M, Popplewell LL, Chavez JC, Bachy E, Kato K (2021) Efficacy of Tisagenlecleucel in Adult Patients (Pts) with High-Risk Relapsed/Refractory Follicular Lymphoma (r/r FL): Subgroup Analysis of the Phase II Elara Study. Blood 138:131. doi:[10.1182/blood-2021-145025](https://doi.org/10.1182/blood-2021-145025)

3. Bartlett NL, Costello BA, LaPlant BR, Ansell SM, Kuruvilla JG, Reeder CB, Thye LS, Anderson DM, Krysiak K, Ramirez C, Qi J, Siegel BA, Griffith M, Griffith OL, Gomez F, Fehniger TA (2018) Single-agent ibrutinib in relapsed or refractory follicular lymphoma: a phase 2 consortium trial. Blood 131 (2):182-190. doi:10.1182/blood-2017-09-804641

4. Metzner B, Pott C, Muller TH, Casper J, Kimmich C, Petershofen EK, Renzelmann A, Rosien B, Thole R, Voss A, Kohne CH, Wellnitz D (2021) Long-term outcome in patients with follicular lymphoma following high-dose therapy and autologous stem cell transplantation. Eur J Haematol 107 (5):543-552. doi:10.1111/ejh.13691

5. Montoro J, Chorao P, Bento L, Cabrero M, Martin C, Novelli S, Cadenas IG, Gutierrez G, Lopez-Godino O, Ferra C, Bastos-Oreiro M, Perez A, Parody R, Perez Simon JA, Yanez L, Sanchez A, Zanabili J, Varela MR, Cordoba R, Zudaire T, Jimenez-Ubieto A, Sanz J, Sureda A, Caballero D, Pinana JL, Geth, Group G (2021) Risk factors and outcomes of follicular lymphoma after allogeneic hematopoietic stem cell transplantation using HLA-matched sibling, unrelated, and haploidentical-related donors. Bone Marrow Transplant 56 (4):992-996. doi:10.1038/s41409-020-01161-1

6. Sakurai M, Mori T, Kato K, Kanaya M, Mizuno S, Shiratori S, Wakayama T, Uchida N, Kobayashi H, Kubo K, Amano I, Ohta T, Miyazaki Y, Kanda J, Fukuda T, Atsuta Y, Kondo E, Adult Lymphoma Working Group of the Japan Society for Hematopoietic Cell Transplatation (2021) Outcome of allogeneic hematopoietic stem cell transplantation for follicular lymphoma relapsing after autologous transplantation: analysis of the Japan Society for Hematopoietic Cell Transplantation. Bone Marrow Transplant 56 (6):1462-1466. doi:10.1038/s41409-020-01192-8

7. Szlauer‐Stefańska A, Sawicki W, Paszkiewicz‐Kozik E, Romejko‐Jarosińska J, Czerw T, Giebel S (2021) Autologous Hematopoietic Stem Cell Transplantation in Follicular Lymphoma in the Era of Novel Therapies-A Retrospective Analysis by Polish Lymphoma Research Group. Hematological Oncology 39. doi:[10.1002/hon.24_2881](https://doi.org/10.1002/hon.24_2881)

8. Mozas P, Sorigué M, Rivero A, Rivas-Delgado A, Bataller A, Giné E, Baumann T, Oliver A, Nadeu F, González-Farré B, Balagué O, Giné E, Baumann T, Delgado J, Villamor N, Campo E, Magnano L, Sancho JM, López-Guillermo A (2020) The interval between frontline treatment and second relapse (PFS2) predicts survival from second relapse (SFR2) in follicular lymphoma patients. HemaSphere 4 (S1): 550-551. doi: [10.1097/HS9.0000000000000404](https://doi.org/10.1097/HS9.0000000000000404)

9. Fuji S, Tada Y, Nozaki K, Saito H, Ozawa T, Kida T, Kosugi S, Sugahara H, Ikeda H, Hashimoto K, Karasuno T, Ueda S, Ishikawa J, Shibayama H (2020) A multi-center retrospective analysis of patients with relapsed/refractory follicular lymphoma after third-line chemotherapy. Ann Hematol 99 (9):2133-2139. doi:10.1007/s00277-020-04126-y

10. Sesques P, Bourcier J, Golfier C, Lebras L, Nicolas-Virelizier E, Hacini M, Perrin MC, Voillat L, Bachy E, Traverse-Glehen A, Moreau A, Martin L, Ramla S, Casasnovas O, Le Gouill S, Salles G, Ghesquieres H (2020) Clinical characteristics and outcomes of relapsed follicular lymphoma after autologous stem cell transplantation in the rituximab era. Hematol Oncol 38 (2):137-145. doi:10.1002/hon.2713

11. Sesques P, Golfier C, Boursier J, Bachy E, Traverse‐Glehen A, Gouill SL, Casasnovas O, Ghesquieres H, G S (2017) Characteristics and outcomes of relapsed follicular lymphoma after autologous stem cell transplantation in the rituximab era. Hematological Oncology 35:220-221. doi:10.1002/hon.2438

12. Khouri IF, Milton DR, Ledesma C, Erwin WD, Jabbour EJ, Alatrash G, Anderlini P, Bashir Q, Ciurea SO, Im JS (2018) Durability Results of Non-Myeloablative (NMA) Allogeneic Stem Cell Transplantation (alloSCT) for Relapsed Follicular Lymphoma: 17-Year Experience. Blood 132:4651. doi:[10.1182/blood-2018-99-117767](https://doi.org/10.1182/blood-2018-99-117767)

13. Smith SM, Godfrey J, Ahn KW, DiGilio A, Ahmed S, Agrawal V, Bachanova V, Bacher U, Bashey A, Bolaños-Meade J (2018) Autologous versus allogeneic transplantation in follicular lymphoma patients experiencing early treatment failure. Cancer 124 (12):2541. doi:[10.1002/cncr.31374](https://doi.org/10.1002/cncr.31374)

14. Sureda A, Zhang MJ, Dreger P, Carreras J, Fenske T, Finel H, Schouten H, Montoto S, Robinson S, Smith SM, Boumedil A, Hamadani M, Pasquini MC (2018) Allogeneic hematopoietic stem cell transplantation for relapsed follicular lymphoma: A combined analysis on behalf of the Lymphoma Working Party of the EBMT and the Lymphoma Committee of the CIBMTR. Cancer 124 (8):1733-1742. doi:10.1002/cncr.31264

15. Sureda A, Zhang M-J, Dreger P, Carreras J, Fenske T, Finel H, Schouten H, Montoto S, Robinson S, Smith S Allogeneic Transplantation for Relapsed/Refractory (R/R) Follicular Lymphoma (Fl). A Joint Study Between the European Society for Blood and Marrow Transplantation (EBMT) and the Center for International Blood and Marrow Transplant Research (CIBMTR). In: Bone Marrow Transplantation, 2016. Nature Publishing Group, pp S68-S68. doi:10.1038/bmt.2016.46

16. Robinson SP, Boumendil A, Finel H, Schouten H, Ehninger G, Maertens J, Crawley C, Rambaldi A, Russell N, Anders W, Blaise D, Yakoub-Agha I, Ganser A, Castagna L, Volin L, Cahn JY, Montoto S, Dreger P (2016) Reduced intensity allogeneic stem cell transplantation for follicular lymphoma relapsing after an autologous transplant achieves durable long-term disease control: an analysis from the Lymphoma Working Party of the EBMTdagger. Ann Oncol 27 (6):1088-1094. doi:10.1093/annonc/mdw124
